# Molecular Mechanisms Underlying the Acclimation of *Chlamydomonas reinhardtii* Against Nitric Oxide Stress

**DOI:** 10.3389/fpls.2021.690763

**Published:** 2021-08-05

**Authors:** Eva YuHua Kuo, Tse-Min Lee

**Affiliations:** ^1^Department of Marine Biotechnology and Resources, National Sun Yat-sen University, Kaohsiung, Taiwan; ^2^Doctoral Degree Program in Marine Biotechnology, National Sun Yat-sen University, Kaohsiung, Taiwan

**Keywords:** acclimation, antioxidant defense system, *Chlamydomonas reinhardtii*, membrane trafficking system, nitrogen homeostasis, nitrosative stress, photosynthesis, sulfur homeostasis

## Abstract

The acclimation mechanism of *Chlamydomonas reinhardtii* to nitric oxide (NO) was studied by exposure to *S*-nitroso-*N*-acetylpenicillamine (SNAP), a NO donor. Treatment with 0.1 or 0.3 mM SNAP transiently inhibited photosynthesis within 1 h, followed by a recovery, while 1.0 mM SNAP treatment caused irreversible photosynthesis inhibition and mortality. The SNAP effects are avoided in the presence of the NO scavenger, 2-(4-carboxyphenyl)-4,4,5,5-tetramethylimidazoline-l-oxyl-3-oxide (cPTIO). RNA-seq, qPCR, and biochemical analyses were conducted to decode the metabolic shifts under NO stress by exposure to 0.3 mM SNAP in the presence or absence of 0.4 mM cPTIO. These findings revealed that the acclimation to NO stress comprises a temporally orchestrated implementation of metabolic processes: (1). modulation of NADPH oxidase (respiratory burst oxidase-like 2, RBOL2) and ROS signaling pathways for downstream mechanism regulation, (2). trigger of NO scavenging elements to reduce NO level; (3). prevention of photo-oxidative risk through photosynthesis inhibition and antioxidant defense system induction; (4). acclimation to nitrogen and sulfur shortage; (5). attenuation of transcriptional and translational activity together with degradation of damaged proteins through protein trafficking machinery (ubiquitin, SNARE, and autophagy) and molecular chaperone system for dynamic regulation of protein homeostasis. In addition, the expression of the gene encoding NADPH oxidase, RBOL2, showed a transient increase while that of RBOL1 was slightly decreased after NO challenge. It reflects that NADPH oxidase, a regulator in ROS-mediated signaling pathway, may be involved in the responses of *Chlamydomonas* to NO stress. In conclusion, our findings provide insight into the molecular events underlying acclimation mechanisms in *Chlamydomonas* to NO stress.

## Introduction

Nitric oxide (NO), a gaseous bioactive free radical that can be enzymatically generated in plants by either NO synthase (NOS) or nitrate reductase (NR), or through non-enzymatic reactions (Besson-Bard et al., 2008; Palavan-Unsal and Arisan, 2009), is a crucial signaling molecule in the regulation of plant growth and development (Anbar, 1995; Hasanuzzaman et al., 2010) and stress responses (Neill et al., 2008; Hayat et al., 2010; Siddiqui et al., 2011). For example, NO signals are involved in stomatal closure (Neill et al., 2002; Guo et al., 2003), the activation of mitogen-activated protein kinase (MAPK) (Zhang et al., 2007), lateral root development regulation and the control of genes associated with the cell cycle (Correa-Aragunde et al., 2004). Nitric oxide pre-treatment not only increases ROS scavenging enzyme activities but also enhances the expression of stress-related genes encoding sucrosephosphate synthase, Δ^1^-pyrroline-5-carboxylate synthase and small heat shock protein 26 (Uchida et al., 2002).

Nitric oxide (NO) is also generated in algae (Mallick et al., 2002; Sakihama et al., 2002; Estevez and Puntarulo, 2005; Ross et al., 2006; Zhang et al., 2006; Chung et al., 2008; Bouchard and Yamasaki, 2009; Chang et al., 2013) and plays a role in the regulation of cell growth and the responses to stress (Murthy et al., 1986; Zhang et al., 2006; Bouchard and Yamasaki, 2009; Lehner et al., 2009; Stephanie et al., 2009; Kuo et al., 2020a). Nitric oxide has been proposed to play a role in the adaptation of an intertidal green macroalga *Ulva lactuca* to desiccation stress (Murthy et al., 1986). The high light-induced NO burst is involved in the regulation of methionine sulfoxide reductase A (MSRA) and MSRB gene expression in *Ulva fasciata* for the regulation of high light acclimation (Hsu and Lee, 2012).

In the green microalga *Chlamydomonas reinhardtii* NO regulates many physiological processes and stress responses such as the remodeling of chloroplast proteins by the degradation of thylakoid cytochrome *b*_6_*f* complex and stroma ribulose-1,5-bisphosphate carboxylase/oxygenase (Rubisco) via FtsH and Clp chloroplast proteases under nitrogen (Wei et al., 2014) or sulfur (de Mia et al., 2019) starvation condition. Nitric oxide is also involved in *Chlamydomonas* cell death induced by ethylene and mastoparan (Yordanova et al., 2010), induction of oxidative stress under extreme high light (VHL, 3,000 μmol⋅m^–2^⋅s^–1^) (Chang et al., 2013), interaction of NO with hydrogen peroxide (H_2_O_2_) for high light stress-induced autophagy and cell death (Kuo et al., 2020a), proline biosynthesis under copper stress (Zhang et al., 2008), and responses to salt stress (Chen et al., 2016). Furthermore, NO is a negative signal for the regulation of nitrogen assimilation by repressing the expression of nitrate reductase (NR) as well as high-affinity nitrate/nitrite transporters and ammonium transporters (de Montaigu et al., 2010) and their enzyme activities (Sanz-Luque et al., 2013; Calatrava et al., 2017). NR, a role for NO synthesis in plants (Calatrava et al., 2017), is involved in the initiation of the pathway for the conversion of nitrite (NO_2_^–^) into NO and then the conversion of NO to nitrate (NO_3_^–^) by the truncated hemoglobin (THB1). Besides, the mitochondrial respiration by upregulation of alternative oxidase 1 is modulated by NO in *Chlamydomonas* (Zalutskaya et al., 2017).

Nitric oxide (NO) protects plants against stress damage (Hasanuzzaman et al., 2010) whereas at higher levels it causes membrane breakdown, DNA fragmentation, and finally cell death (Pedroso et al., 2000; Yamasaki and Sakihama, 2000; Romero-Puertas et al., 2004). Thus, at different concentrations, NO either promotes or inhibits cell death (Delledonne et al., 2001). Nitric oxide also exhibits both beneficial and harmful roles under stressful conditions, depending on its concentration as well as tissue, age, or physiological status of the plants, the ability of NO to interact with other signaling molecules, and the type of stress involved (Arasimowicza and Floryszak-Wieczorek, 2007). Our previous works have demonstrated that NO over-produced in *Chlamydomonas* cells under 3,000 μmol⋅m^–2^⋅s^–1^ condition is involved in the oxidative damage due to the inhibition of carotenoid synthesis and photosynthetic activity (Chang et al., 2013). Recently, we have discovered that NO is associated with the induction of ATG gene expression and the increase of ATG8 protein abundance for the regulation of autophagy in *Chlamydomonas* cells in response to high light (HL, 1,600 μmol.m^–2^.s^–1^) condition (Kuo et al., 2020a). The treatment of two NO donors, S-nitroso-N-acetylpenicillamine (SNAP) and S-nitrosoglutathione (GSNO), under normal light condition (NL, 50 μmol.m^–2^.s^–1^) also increases ATG transcript abundance and ATG8 protein level and triggers cell death in the presence of H_2_O_2_ (Kuo et al., 2020a). It suggests that NO can promote the susceptibility of *C. reinhardtii* cells to high intensity illumination, which results in ROS over-production. By applying SNAP or GSNO at concentrations of 0.05 or 0.1 mM under a moderate high light condition (ML, 750 μmol.m^–2^.s^–1^), the impact on cell growth and viability increases as the concentration of NO donors increases from 0.05 to 0.1 mM (Kuo et al., 2020a). However, information is still lacking on the comprehensive overview of metabolic shifts of *C*. *reinhardtii* in the acclimation to NO stress. Here, SNAP was exogenously administrated in low (0.1 and 0.3 mM), moderate (0.7 mM), and high (1 mM) concentrations in the presence or absence of an NO scavenger, 2-(4-carboxyphenyl)-4,4,5,5-tetramethylimidazoline-l-oxyl-3-oxide (cPTIO) (Mur et al., 2013) under NL condition to avoid other interferences. Treatment with 1 mM SNAP will lead to significant cell death, while 0.7 mM SNAP treatment caused an approximately 50% inhibition on cell growth (after 24 h of culture) and 0.3 mM SNAP treatment triggered a around 26% growth inhibition. The treatment with 0.7 mM SNAP is considered a more serious NO stress as compared to 0.3 mM SNAP treatment.

Because the unicellular green alga *C. reinhardtii* is an excellent genetic and genomic model species for studying a broad range of essential biological processes including the evolution of chloroplast-based photosynthesis (Rochaix, 1995), many well-established *C. reinhardtii* genome sequences and molecular tools are available (Lopez et al., 2011; Goodstein et al., 2012). It provides an opportunity to explore the large-scale molecular processes by comparative genomics coupled with transcriptomic and molecular function analysis. *Chlamydomonas reinhardtii* responses to varying environmental conditions can be best evaluated by using different transcriptomic applications, e.g., illumina analysis or microarray, during lipid accumulation (Lv et al., 2013), copper nutrition deficiency (Castruita et al., 2011), dark anoxia (Hemschemeier et al., 2013), sulfur starvation (Nguyen et al., 2008), nitrogen deprivation (Miller et al., 2010), or CO_2_ deprivation (Brueggeman et al., 2012). These results indicate that genome-wide analysis can in principle be used to discover genes involved in entire responsive molecular events. Here, the present experiment has explored the key metabolic changes caused by NO stress and its possible action mechanism for gaining a full picture of acclimation machinery in *Chlamydomonas* cells to NO stress. First, the transcriptome analysis in response to 0.3 mM SNAP treatment was applied to unravel the acclimation mechanisms underlying NO functions. By next generation sequencing (NGS) of the Illumina technology, a huge number of distinct gene expressions from NO-treated *C. reinhardtii* cells at early phase (1 h) were obtained. Next, the time-course changes in the expression of significantly expressed genes were determined from 0, 0.5, 1, 1.5, 3, and 6 h after exposure to SNAP or GSNO for the comparison with physiological and biochemical data. Furthermore, the extent of the expression of genes was examined in response to serious NO stress (0.7 mM SNAP) for the confirmation of potential mechanisms involving in the acclimation machinery to coping with NO stress. The present findings provide new insight into the acclimation mechanisms against NO stress in *Chlamydomonas*.

## Materials and Methods

### Algal Culture and Chemical Treatments

The green alga *Chlamydomonas reinhardtii*, strain CC-125 (mt-), was obtained from the Chlamydomonas Resource Center (United States) and photoheterotrophically cultured in Tris-acetate phosphate medium (TAP) (Harris, 1989) with a trace element solution in 125 mL flasks (PYREX, Germany) and agitated on an orbital shaking incubator (model OS701, TKS company, Taipei, Taiwan) (150 rpm) under continuous illumination with white light (50 μmol⋅m^–2^⋅s^–1^) at 25°C. For chemical treatments, 50 mL cultures were grown to a cell density of 3–5 × 10^6^ cells⋅mL^–1^, and after centrifugation at 1,600 × *g* for 3 min, the supernatant was discarded. The pellet was suspended in fresh TAP medium and centrifuged again. Then, the pellet was re-suspended in fresh TAP medium to a cell density of 3 × 10^6^ cells⋅mL^–1^. Ten milliliters of culture were transferred to a 100-mL beaker (internal diameter: 3.5 cm) for pre-incubation at 25°C in 50 μmol⋅m^–2^⋅s^–1^ conditions for 1.5 h in an orbital shaker (model OS701, TKS company, Taipei, Taiwan) at a speed of 150 rpm. Then, the algal cells were subjected to treatments at 25°C. SNAP or GSNO was treated in different concentrations from 0.1, 0.3, 0.7, to 1.0 mM in the presence or absence of 0.4 mM cPTIO. Dimethyl sulfoxide (DMSO) was used as the control because SNAP or GSNO was dissolved in DMSO. Each treatment included three biological independent replicates (*n* = 3). For the determination of cell growth and the estimation of several physiological and biochemical parameters, the number of cells in a 1-mL sample was counted using a hemocytometer. Samples taken before (0 min) and after treatment were centrifuged at 5,000 × *g* for 5 min, and the pellet was fixed in liquid nitrogen and stored in a −70°C freezer until analysis.

### Detection of NO Flux

An NO-sensitive fluorescent dye, DAF-FM diacetate (Invitrogen Life Technologies, Carlsbad, CA, United States) (Kojima et al., 1998), was used to measure NO production following our previous studies (Kuo et al., 2020b). DAF-FM diacetate is a pH-insensitive fluorescent dye that emits fluorescence after reaction with an active intermediate of NO (Kojima et al., 1998; Chang et al., 2013). The cells were pre-incubated in TAP medium containing 5 μM DAF-FM diacetate for 60 min at 25°C under 50 μmol⋅m^–2^⋅s^–1^ conditions, then washed twice with fresh TAP medium, and transferred to 50 μmol⋅m^–2^⋅s^–1^ for chemical treatment. Besides, before fluorescence detection, the cells were washed twice with fresh TAP medium again. The fluorescence was detected via fluorescence microscopy and spectrophotometry. Because the fluorescence spectrophotometry is relatively sensitive than fluorescence microscopy, the basal DAF-FM fluorescence value of 2.18-3.42 can be measured in the control samples over 0–6 h period, although it is not visualized under fluorescence microscopy. The level of DAF-FM fluorescence determined in this study represented the cumulative NO production because the DAF-FM dye was loaded prior to the treatment. Thus, the NO flux rate can be estimated from the difference in relative fluorescent units (RFU) between the two time points. Based on 10^6^ cells, DAF-FM fluorescence flux rate was expressed as RFU.h^–1^.

The NO flux was also determined using Griess method according to the reduction of NO_3_^–^ to NO_2_^–^ and the determination of NO_2_^–^ the Griess reaction based on a two-step diazotization reaction in which the NO-derived nitrosating agent, dinitrogen trioxide (N_2_O_3_) generated from the acid-catalyzed formation of nitrous acid from NO_2_^–^ (or autoxidation of NO) reacts with sulfanilamide to produce a diazonium ion which is then coupled to N-(1-napthyl)ethylenediamine to form a chromophoric azo product that absorbs strongly at 540 nm (Grisham et al., 1996).

### Determination of Cell Density and Viability

For cell density estimation, 10 μL of algal culture was mixed with 30 μL of Lugol’s solution (Sigma-Aldrich, St. Louis, MO, United States) and the cell number was counted in duplicate using a light microscope (BX43, Olympus, Tokyo, Japan) and a hemacytometer (Improved Neubauer, Boeco, Germany) as mentioned above. The cell density was calculated according to the manufacturer’s manual and expressed as units of 10^6^**⋅**mL^–1^.

After 6 h of treatments, the viability of algal cells was estimated by loading 2 μL of cell suspension on TAP agar plates, and incubating the plates for 72 h at 28°C under illumination at 50 μmol**⋅**m^–2^**⋅**s^–1^ intensity. The colonies were imaged with a digital Nikon camera, and the final composite images were constructed using Adobe Photoshop (Adobe Systems, San Jose, CA, United States). Together with cell density curve, the cell viability assessed by growth ability (the color and size of the colony) was used to evaluate the effects of chemical challenges.

### Detection of Dead Cells Using SYTOX Green Fluorescence

Cell death was assessed using the SYTOX-Green fluorescent probe (Molecular Probes Inc., Eugene, OR, United States). The SYTOX-Green stock solution of 5 mM in 100% DMSO was added to 1 mL of algal culture at a final concentration of 5 μM, and the mixture was incubated for 5 min at room temperature in the dark. The fluorescence level was detected by a fluorescence spectrophotometer at 525 nm (excitation: 488 nm) (Sato et al., 2004). Based on the blank (TAP medium without algal cells), the relative SYTOX-Green fluorescence level was estimated and expressed as relative fluorescence**⋅**(10^6^ cells)^–1^. Then, the cells were observed with a fluorescence microscope (Eclipse Ni, Nikon, Tokyo, Japan) with excitation at 488 nm using Nikon Fluorescein isothiocyanate (FITC) (excitation wavelength: 465–495 nm, emission wavelength: 515–555 nm) and B-2A (excitation wavelength: 450–490 nm, emission wavelength: >520 nm) fluorescence filters (Nikon, Tokyo, Japan). Fluorescent images were acquired using a Charge-coupled Device (CCD) camera (Nikon’s Digital Sight DSU3, Tokyo, Japan) and imported into Adobe Photoshop.

### Determination of Chlorophyll *a* Fluorescence

Chlorophyll *a* fluorescence parameters were employed to determine the activity of photosystem II (PSII) using an AP-C 100 (AquaPen, Photon Systems Instruments, Brno, Czech Republic). A 0.5-mL aliquot of an algal culture was diluted with TAP medium to an OD_750_ = 0.10–0.15 with a chlorophyll *a* content of 1.5–2.2 μg⋅mL^–1^. A 2-mL aliquot of diluted algal cells was then transferred to an AquaPen cuvette and subjected to a pulse of saturating light of 4,000 μmol⋅m^–2^⋅s^–1^ PAR to obtain the light-adapted minimal fluorescence (*F*_*t*_) and the light-adapted maximal fluorescence (*F*_*m*_’). To determine the maximum PSII activity, *F*_*v*_/*F*_*m*_ (= *F*_*m*_ – *F*_*o*_/*F*_*m*_), 2 mL of diluted algal cells in an AquaPen cuvette was incubated in the dark for 20 min and then flushed with saturated light (4,000 μmol photons⋅m^–2^⋅s^–1^) to obtain the dark-adapted minimal fluorescence (*F*_*o*_) and dark-adapted maximal fluorescence (*F*_*m*_). The active PSII activity, *F*_*v*_’/*F*_*m*_’ = *F*_*m*_’ – *F*_*t*_/*F*_*m*_’, and the maximum PSII activity, *F*_*v*_/*F*_*m*_ = *F*_*m*_– *F*_*o*_/*F*_*m*_, were then calculated.

The rapid induction of chlorophyll *a* fluorescence was also determined via the OJIP test (Strasser and Strasser, 1995; Strasser et al., 2000). The inflections in the O-J-I-P curve represent the heterogeneity of the process during photochemical action; peak J represents the momentary maximum level of Q_*A*_^–^, Q_*A*_^–^Q_*B*_, and Q_*A*_^–^Q_*B*_^–^; peak I represents the level of Q_*A*_^–^Q_*B*_^2–^; and peak P represents the maximum level of Q_*A*_^–^, Q_*B*_^2–^, and PQH_2_ (Stirbet et al., 1998). The fluorescence values at time intervals corresponding to the O-J-I-P peaks were recorded as follows: *F*_*o*_ = fluorescence intensity at 50 μs; *F*_*J*_ = fluorescence intensity at peak J (at 2 ms); *F*_*i*_ = fluorescence intensity at peak I (at 60 ms); *F*_*m*_ = maximal fluorescence intensity at the peak P; *F*_*v*_ = *F*_*m*_ – *F*_*o*_ (maximal variable fluorescence); V_*j*_ = (*F*_*j*_ – *F*_*o*_)/(*F*_*m*_ – *F*_*o*_); V_*i*_ = (*F*_*i*_ – *F*_0_)/(*F*_*m*_ – *F*_*o*_); and *F*_*m*_/*F*_*o*_; *F*_*v*_/*F*_*o*_; *F*_*v*_/*F*_*m*_.

### Determination of Photosynthetic O_2_ Evolution Rate and Respiration Rate

The amount of O_2_ released or uptake by algal cells were detected using a Clark-type oxygen electrode fitted with a DW3 chamber (Hansatech, Kings Lynn, Norfolk, United Kingdom) with temperature controlled at 25°C using a thermostat. For sampling, the cells were collected and centrifuged at 5000 × *g* for 10 min and then the cell pellet was suspended in 50 mM 4-(2-hydroxyethyl)-1-piperazineethanesulfonic acid (HEPES) buffer (pH 7.4) with a density of 1.0 × 10^6^ cells⋅mL^–1^ containing 5 mM NaHCO_3_. Then, the photosynthetic O_2_ evolution was determined at a light intensity of 800 μmol⋅m^–2^⋅s^–1^ till stable slope appeared. After that, light was turned off to determine respiratory O_2_ uptake rate. Because the preliminary test showed that photosynthetic O_2_ evolution reached plateau during 600–1,400 μmol photons⋅m^–2^⋅s^–1^, followed by a slight drop during 1,600–2,000 μmol ⋅m^–2^⋅s^–1^, the intensity of 800 μmol photons⋅m^–2^⋅s^–1^ was chosen for the determination of photosynthetic O_2_ evolution rate. The light source used was low voltage Tungsten halogen lamps (12 V, 50 W; Sylvania, Danvers, MA, United States). Three replicates per treatment were performed. The net photosynthetic O_2_ evolution rate and the respiration rate were expressed as μmol O_2_ evolution⋅h^–1^⋅10^–6^ cell and μmol O_2_ uptake⋅h^–1^⋅10^–6^ cell, respectively. The gross photosynthetic O_2_ evolution rate was the sum of net photosynthetic O_2_ evolution rate and the respiration rate.

### Enzyme Activity Assay and Determination of AsA and GSH

In-gel superoxide dismutase (SOD) activity was assayed according to Page et al. (2012). Glutathione reductase (GR) and ascorbate peroxidase (APX) activity was determined according to the methods described by Lin et al. (2018) and Kuo et al. (2020b), respectively. Protein concentrations were quantified using the Coomassie Blue dye binding method (Bradford, 1976) using a concentrated dye purchased from BioRad (500–0006, Hercules, CA, United States). Ascorbate (AsA) and dehydroascorbate (DHA) concentrations were determined by an ascorbate oxidase based method according to Lin et al. (2020). GSH and oxidized glutathione (GSSG) were extracted from the frozen algal cell pellet (obtained from 5 mL samples) using 5% (w/v) trichloroacetic acid (TCA) and determined at 412 nm according to Lin et al. (2018).

### RNA Isolation, cDNA Synthesis, Transcriptomic Analysis, and mRNA Quantification *via* Real-Time Quantitative PCR

Total RNA was extracted using the TriPure Isolation Reagent (Roche Applied Science, Mannheim, Germany) according to the manufacturer’s instructions. The methods for cDNA library preparation, Illumina sequencing, and sequence analysis are described in Supplementary Figure 6. For qPCR assay, the total RNA concentration was adjusted to 2.95 μg total RNA⋅μL^–1^ and treated with DNase (TURBO DNA-free^TM^ Kit, Ambion Inc., The RNA Company, United States) to remove residual DNA. Then 1.5 μg of total RNA was used for the preparation of cDNA. cDNA was amplified from the poly-(A +) tail using Oligo (dT)12–18 with the VersoTM cDNA Kit (Thermo Fisher Scientific Inc., Waltham, MA, United States), and the volume was adjusted to a concentration of 30 ng⋅mL^–1^ based on original RNA quantity in each sample. The primers for the targeted genes are listed in Supplementary Table 1. The real-time quantitative PCR was performed using a LightCycler 480 system (Roche Applied Science, Mannheim, Germany). A PCR master mix was prepared with the LightCycler 480 SYBR Green I Master Kit (Roche Applied Science, Mannheim, Germany). Each reaction was performed in a total volume of 10 μL, containing 1 × LightCycler 480 SYBR Green I Master Mix, the selected concentration of each primer, and cDNA corresponding to 30 or 50 ng⋅μL^–1^ RNA in the reverse transcriptase reaction. The amplification program consisted of an initial denaturation at 95°C for 5 min, followed by 50 amplification cycles of annealing at 60°C for 10 s, elongation at 72°C for 5 s, real-time fluorescence measurements, and finally, denaturation at 95°C for 15 s. The 2^–ΔΔ^CT method was used to calculate the relative change in mRNA level normalized to a reference gene, ubiquitin-conjugating enzyme E2 isoform (UBC, NCBI: AY062935) and the fold increase was calculated relative to the control RNA sample at 0 min. Because the results based on the elongation factor 1 alhpa (EF-1α, NCBI: XM_001696516.1) internal control were similar to those based on UBC, the relative changes in the levels of gene transcription were expressed based on UBC.

### Western Blots

Soluble protein was extracted according to Kuo et al. (2020a). For each sample, 30 μg of protein was loaded into each lane, resolved on a 15% sodium dodecyl sulfate polyacrylamide gel electrophoresis (SDS-PAGE) gel, and transferred to a polyvinylidene fluoride membrane for antibody binding with rabbit polyclonal anti-ATG8 antibody (ab77003; Abcam, Cambridge, United Kingdom), anti-DHAR (LKT BioLaboratories Ltd., Taoyuan, Taiwan), or a mouse monoclonal antibody against α-tubulin (ab11304; Abcam, Cambridge, United Kingdom). Following the incubation with horseradish peroxidase-conjugated secondary antibodies (MD20878; KPL, Gaithersburg, MD, United States), the immunoblots were visualized and the relative abundance of ATG8, ATG8-PE, or DHAR1 protein was estimated based on α-tubulin intensity.

### Statistics

Three independent biological replicates were performed and all experiments were repeated at least three times. Because the replications showed similar trends, only the results from one replicate were shown in this paper. Statistical analyses were performed using SPSS (SPSS 15.0, Chicago, IL, United States). Significant differences between means were analyzed using Student’s *t*-test or Scheffe’s test following significant analysis of variance for the controls and treatments at *P* < 0.05.

## Results

### Physiological and Transcriptomic Changes in Response to NO Burst

**FIGURE 1:**
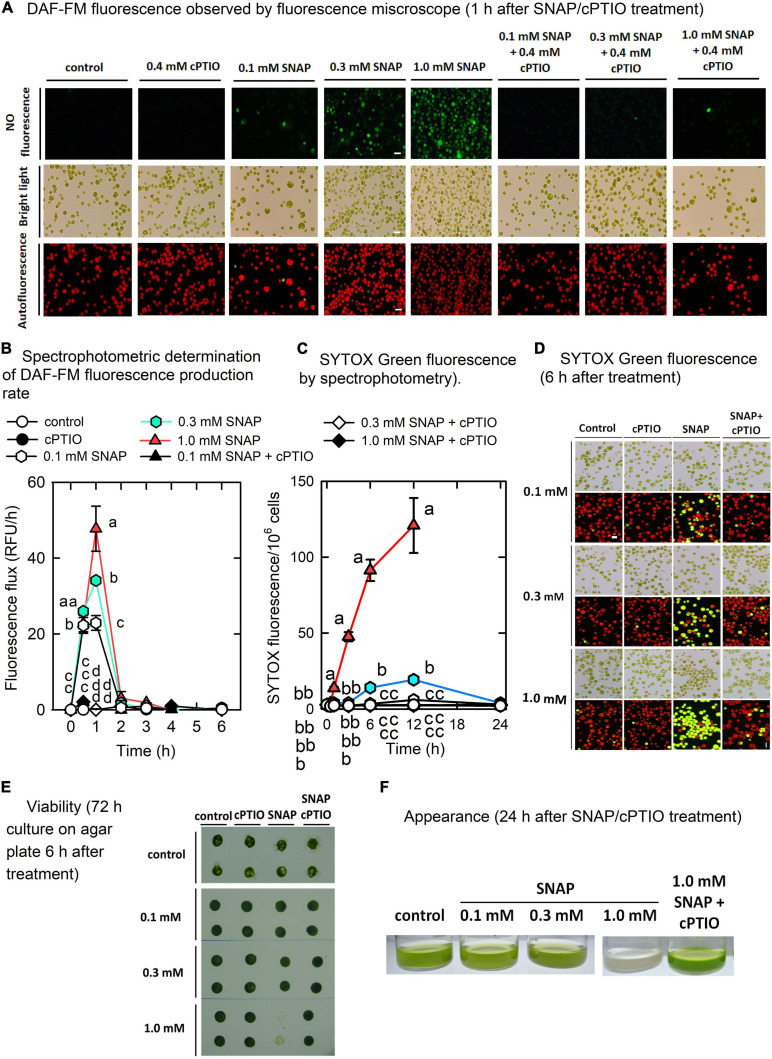
Physiological response to NO. Microscopic observation **(A)** and spectrophotometric determination **(B)** of nitric oxide fluorescence (DAF-FM), spectrophotometric quantitative determination of SYTOX Green fluorescence **(C)**, microscopic observation of SYTOX green staining **(D)**, cell viability **(E)**, and appearance **(F)** in *Chlamydomonas reinhardtii* in response to 0.1, 0.3, or 1.0 mM SNAP in the presence or absence of 0.4 mM cPTIO. For cell viability assay, the cells were treated with chemicals for 6 h and then transferred to agar plate for another 72 h. Data are expressed as the mean ± SD (*n* = 3). Different symbols indicate significant differences between treatments (Scheffe’s test, *P* < 0.05).

The mechanisms that allow for the acclimation of *C*. *reinhardtii* cells to short-term NO burst were examined. The SNAP concentration and treatment duration were carefully chosen to allow for the monitoring of the short-term response to NO rather than cell death. Using a cell permeable NO-sensitive fluorescent dye, the fluorescence emitted from the cells (Figure 1A) rapidly increased 0.5 h after SNAP treatment and reached a plateau after 1 h (Figure 1B); the increase in fluorescence can be inhibited in the presence of 0.4 mM cPTIO. However, NO is unevenly distributed in cells under 0.1 mM SNAP treatment, which some cells exhibit DAF-FM fluorescence while some are not (Figure 1A). It indicates that heterogeneous cells with different entrance ability of NO into inner cellular space are existing in *Chlamydomonas* cells mixtrophically cultured in TAP medium. Thus, the cells in response to 0.1 mM SNAP were not used for the following experiments. The level of DAF-FM fluorescence determined in this study represented the cumulative NO production because the DAF-FM dye was loaded prior to the treatment. Thus, the NO production rate can be estimated from the difference in relative fluorescent units (RFU) between the two time points. Since the fluorescence production rate reached a peak after 1 h and then a fast drop to the control level after 2 h, it indicates that NO is not or less released from SNAP after 2 h. Using Griess method, NO was generated fast after SNAP exposure within 1 h and then released in a small amount after 2 h (Supplementary Figure 1). In addition to SNAP, another NO donor, GSNO These results indicate that NO bursts within 1 h after treatment of NO donor. Based on the above two methods, the amount of NO emitted from GSNO applied in the TAP medium showed a similar trend as SNAP treatment (Supplementary Figures 2B,C).

SNAP treatments affected cell viability (Figures 1C–E), in which as SNAP concentration increased, viability decreased while the 1.0 mM SNAP treatment resulted in significant cell mortality after 12 h, followed by a complete cell death (Figure 1E) and bleaching (Figure 1F). Using SYTOX Green staining of dead cells, the estimation of the SYTOX Green fluorescence showed that the cells emiting red autofluorescence without yellow fluorescence (yellow fluorescence was the merge of red and green fluorescence) are 18.56 ± 3.19% after 6 h for 1.0 mM SNAP treatment, then down to 8.63 ± 2.51% after 12 h, and there were no cells can be found for 24 h. Furthermore, by the estimation of the number of cells including those intact ones without lysis that have been stained with SYTOX Green fluorescence, the density of the cells treated with 0.1 mM SNAP was smaller than that of the control after 24 h (7.10 × 10^6^ cells/mL compared to 8.12 × 10^6^ cells/mL in the control), while that treated with 0.3 mM SNAP showed a 26.36% decrease (5.98 × 10^6^ cells/mL as compared to 8.12 × 10^6^ cells/mL in the control) (data not shown). The cell number of 1.0 mM SNAP treatment was significantly decreased in a short time to 0.85 × 10^6^ cells/mL after 12 h, followed by the absence of cells after 24 h (data not shown).

**FIGURE 2:**
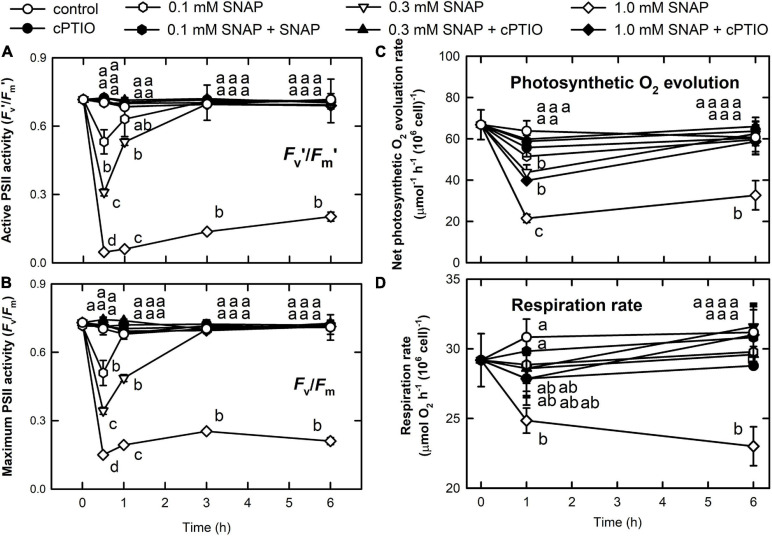
Changes in photosynthetic activity and respiration rate in response to NO. The active (*F*_*v*_’/*F*_*m*_’) **(A)** and maximum (*F*_*v*_/*F*_*m*_) **(B)** PSII activity, photosynthetic O_2_ evolution rate **(C)**, and respiration rate **(D)** in *C. reinhardtii* after exposure to 0.1, 0.3, or 1.0 mM SNAP in the presence or absence of 0.4 mM cPTIO. Data are expressed as the mean ± SD (*n* = 3). Different symbols indicate significant differences between treatments (Scheffe’s test, *P* < 0.05).

The concentrations of chlorophyll *a* (Supplementary Figure 2A), chlorophyll *b* (Supplementary Figure 2B), and carotenoids (Supplementary Figure 2C) were not influenced by either 0.1 or 0.3 mM SNAP treatments; however, a decrease in chlorophyll *a*, chlorophyll *b*, and carotenoids 6 h after treatment with 1.0 mM SNAP was observed. Photosynthesis is relatively sensitive to NO burst, as reflected by a transient decrease in the active PSII activity, *F*_*v*_’/*F*_*m*_’ (Figure 2A), the maximum PSII activity, *F*_*v*_/*F*_*m*_ (Figure 2B), the O_2_ evolution rate (Figure 2C), and the respiration rate (Figure 2D) 0.5 h after SNAP treatment; these photosynthesis-related factors then recovered after 3–5 h in both the 0.1 and 0.3 mM SNAP treatments but not in the 1.0 mM SNAP treatment. This inhibition of photosynthesis-related factors can be suppressed in the presence of 0.4 mM cPTIO. Based on fluorescence induction kinetics (OJIP curve), the cells treated with 0.1 or 0.3 mM SNAP transiently lost both the PSII acceptor-side (*F*_*J*_–*F*_*o*_ and *F*_*I*_–*F*_*J*_) and donor-side (*F*_*P*_–*F*_*I*_ and *F*_*v*_/*F*_*o*_) electron transfer ability, whereas 1.0 mM SNAP treatment caused irreversible inhibition, which was prevented in the presence of 0.4 mM cPTIO (Supplementary Table 2). The SNAP treatments decreased the production of superoxide anion radical (O_2_. ^–^) and H_2_O_2_; the production of singlet oxygen (^1^O_2_) was significantly increased by 1.0 mM SNAP treatment (Supplementary Figures 3D–F). Treatment with 1.0 mM SNAP also caused significant lipid peroxidation (Supplementary Figure 3G). Together with significant oxidative stress, an irreversible inhibition of photosynthetic activity and respiration rate and severe morality when exposed to 1.0 mM SNAP treatment, the SNAP of 0.3 mM in the presence or absence of 0.4 mM cPTIO is chosen for the study of the acclimation of *Chlamydomonas* cells to NO burst. Overall, these results demonstrate two stages of response in *C*. *reinhardtii* cells in the NO challenge (0.3 mM SNAP): I. NO stress together with the significant NO burst caused a sharp decline in photosynthetic activity 1 h after SNAP treatment and II. the recovery period. First, *Chlamydomonas* cells treated with 0.3 mM SNAP for 1 h in the presence or absence of 0.4 mM cPTIO were used for transcriptomic analyses. Then, based on the data of transcriptome analysis, the time-course variation over 0–6 h in biochemical parameters and transcript abundance of selected genes were assayed for the elucidation of acclimation machinery in *Chlamydomonas* cells to NO stress.

The results of the transcriptomic analysis (Supplementary Tables 3, 4) showed that 1,012 significant differentially expressed genes (DEGs) were regulated by NO (1.2 log_2_FC, *P*-value of log_2_FC ≤ 0.05) (Supplementary Table 5). Following analysis using the MapMan display mode, cellular response overview (Supplementary Figure 4), proteasome and autophagy (Supplementary Figure 5), photosynthesis (electron transport, Calvin cycle, and photorespiration) (Supplementary Figure 6), and the tetrapyrrole pathway (Supplementary Figure 7) were affected under NO stress. Using the Blast2GO suite, the analysis of all the functional DEGs and unigenes identified 45 GO terms (score ≤ 0.05) that can be assigned to 463 upregulated genes (45.75%), with 13 biological process, 30 molecular function, and two cellular component Gene Ontology (GO) terms (Supplementary Figure 8A and Supplementary Table 6A). One hundred fifty-seven GO terms for 549 downregulated genes with 337 known function genes (54.25%) were classified into 35 GO terms belonging to biological process, 112 to molecular function, and eight to cellular component (Supplementary Figure 8B and Supplementary Table 6B). The genes associated with amino acid catabolism, the ubiquitin-proteasome system, and the antioxidant defense system are upregulated, while those related to transcriptional and translational regulation are downregulated by NO burst (Supplementary Tables 5, 6).

**FIGURE 3:**
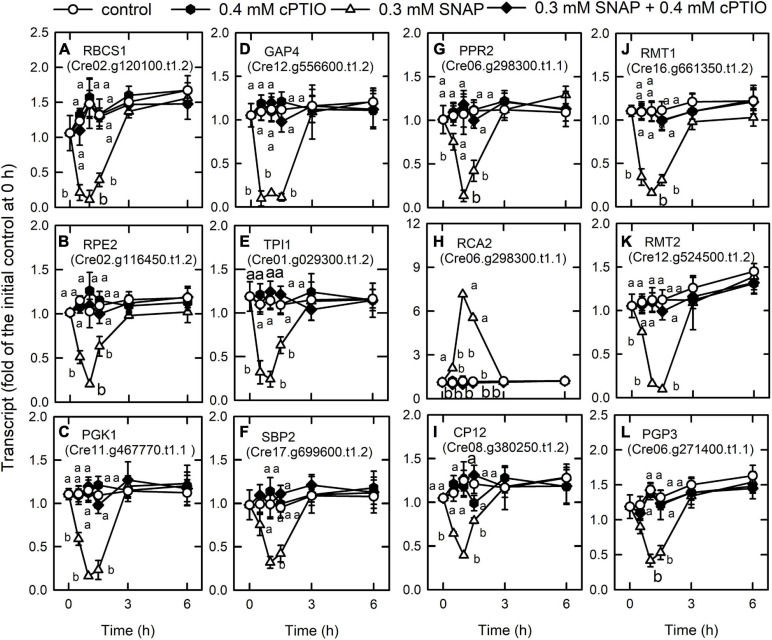
Effects of NO on the expression of genes encoding Calvin cycle proteins. Time-course changes in the transcript abundances of RBCS1 **(A)**, RPE2 **(B)**, PGK1 **(C)**, GAP4 **(D)**, TPI1 **(E)**, SBP2 **(F)**, PPR2 **(G)**, RCA2 **(H)**, CP12 **(I)**, RMT1 **(J)**, RMT2 **(K)**, and PGP3 **(L)** in *C. reinhardtii* after exposure to 0.3 mM SNAP in the presence or absence of 0.4 mM cPTIO. Data are expressed as the mean ± SD (*n* = 3). Different symbols indicate significant differences between treatments (Scheffe’s test, *P* < 0.05).

We also found that the genes associated with photosynthesis are downregulated by NO burst (Supplementary Table 8). NO decreased the transcript abundances of lumenal PsbP-like protein (PSBP4) that is linked to the stability of photosystem II complex assembly (Ido et al., 2014), Lhl2 belonging to high light-induced protein (Teramoto et al., 2004), Lhl3, pre-apoplastocyanin (PCY1), cytochrome c_6__*A*_ (CYC4) acting as electron transfer between cytochrome *b*_6_*f* complex and photosystem I (Marcaida et al., 2006), CHLH, CHLI1, CHLI2, CHLD, and GENOMES UNCOUPLED 4 (GUN4). In contrast, NO increased the transcript abundance of chlorophyll *a*/*b* binding protein (LHCBM9), Lhl1, and Lhl4 (Supplementary Figure 9). For the Calvin cycle, NO also decreased the transcript abundance of the Rubisco small subunit 1 (RBCS1, Cre02.g120100.t1.2), ribulose phosphate-3-epimerase (RPE2, Cre02.g116450.t1.2), phosphoglycerate kinase (PGK1, Cre11.g467770.t1.1), glyceraldehyde-3-phosphate dehydrogenase (GAP4, Cre12.g556600.t1.2), triose phosphate isomerase (TPI1, Cre01.g029300.t1.2), sugar bisphosphatase (SBP2, Cre17.g699600.t1.2), a pentatrichopeptide repeat protein that stabilizes Rubisco large subunit mRNA (PPR2, Cre06.g298300.t1.1), a small protein for phosphoribulokinase deactivation (Howard et al., 2008) (CP12, Cre08.g380250.t1.2), Rubisco large (RMT1, Cre16.g661350. t1.2 and a similar one, Cre16.g649700.t1.1) and small (RMT2, Cre12.g524500.t1.2) subunit N-methyltransferase, and phosphoglycolate phosphatase (PGP3, Cre06.g271400.t1.1) in the photorespiration (Figure 3). In contrast, the transcript abundance of Rubisco activase-like protein (RCA2, Cre06.g298300.t1.1) was increased by NO (Figure 3H). The NO-induced changes in gene expression occurred 1 h after NO exposure, recovered to the same levels as the control after 3 h, and the changes at 1 h can be inhibited by the presence of cPTIO.

### NO Decreases Nitrogen and Sulfur Availability

**FIGURE 4:**
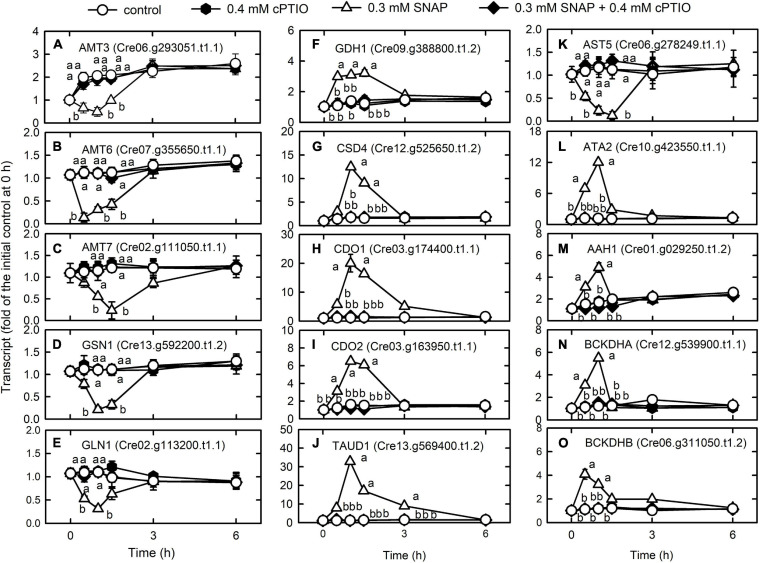
Effects of NO on the expression of genes encoding proteins associated with nitrogen metabolism. Time-course changes in the transcript abundances of AMT3 **(A)**, AMT6 **(B)**, AMT7 **(C)**, GSN1 **(D)** GLN1 **(E)**, GDH1 **(F)**, CSD4 **(G)**, CDO1 **(H)**, CDO2 **(I)**, TAUD1 **(J)**, AST5 **(K)**, ATA2 **(L)**, AAH1 **(M)**, BCKDHA **(N)**, and BCKDHB **(O)** in *C. reinhardtii* in response to 0.3 mM SNAP in the presence or absence of 0.4 mM cPTIO. Data are expressed as the mean ± SD (*n* = 3). Different symbols indicate significant differences between treatments (Scheffe’s test, *P* < 0.05).

The transcript abundances of the ammonium transporters AMT3 (Cre06.g293051.t1.1) (Figure 4A), AMT6 (Cre07.g355650.t1.1) (Figure 4B), and AMT7 (Cre02.g111050.t1.1) (Figure 4C), and the ammonium assimilation enzymes, glutamate synthase (GSN1, Cre13.g592200.t1.2) (Figure 4D) and glutamate synthetase (GLN1, Cre02.g113200.t1.1) (Figure 4E) decreased 1 h after NO treatment and then recovered, while that of glutamate dehydrogenase (GDH1, Cre09.g388800.t1.2) increased after 1 h (Figure 4F).

Cysteine desulfurase (CSD4, Cre12.g525650.t1.2), which moves the sulfur from the cysteine to sulfur-containing recipients, showed an increase in transcriptional expression in the NO treatment (Figure 4G). Cysteine can be oxidized to 3-sulfinoalanine (Chai et al., 2005) by cysteine dioxygenase (CDO), and the transcript abundances of CDO1 (Cre03.g174400.t1.1) (Figure 4H) and CDO2 (Cre03.g163950.t1.1) (Figure 4I) increased after NO treatment. In the NO treatment, the transcript abundance of taurine dioxygenase (TAUD1, Cre13.g569400.t1.2), which degrades taurine to sulfite and aminoacetaldehyde, increased (Figure 4J) but that of aspartate aminotransferase (AST5, Cre06.g278249.t1.1), which converts 3-sulfinoalanine to pyruvate, decreased (Figure 4K). Thus, cysteine is catabolized to taurine and then degraded to sulfite instead of pyruvate. Then, sulfite is oxidized by sulfite oxidase (SUOX) to avoid toxicity due to accumulation (Hansch et al., 2007). However, NO did not affect the expression of SUOX (Cre04.g217929.t1.1) (Supplementary Table 4).

The expression of genes related to amino acid degradation was transiently upregulated by NO, including L-allo-threonine aldolase (ATA2, Cre10.g423550.t1.1) (Figure 4L), the aromatic amino acid hydroxylase-related protein (AAH1, Cre01.g029250.t1.2) (Figure 4M), and the catabolism of branched-chain amino acids (valine, leucine, and isoleucine; 2-oxoisovalerate dehydrogenase E1 component alpha (BCKDHA, Cre12.g539900.t1.1) (Figure 4N) and beta (BCKDHB, Cre06.g311050.t1.2) (Figure 4O) (Supplementary Table 7). The presence of cPTIO inhibited the changes induced by SNAP treatment.

Nitric oxide (NO) increased the transcript abundance of periplasmic arylsulfatase (ARS3), arylsulfatase (ARS7), plasma membrane sodium/sulfate co-transporters (SLT1, SLT2), the chloroplast sulfate binding protein as the component of chloroplast transporter (SULP3), and SNRK2.2 (SAC3), an Snf1-like serine/threonine kinase responsible for the repression of expression of genes responsible for sulfur starvation (Davies et al., 1999; Gonzalez-Ballester et al., 2008). Nitric oxide decreased the transcript abundance of ARS5, ARS11, ARS13, ARS14, and ARS16 (Supplementary Figure 10). The presence of cPTIO inhibited these changes in response to SNAP treatment.

### NO Modulates Protein Homeostasis and Quality

**FIGURE 5:**
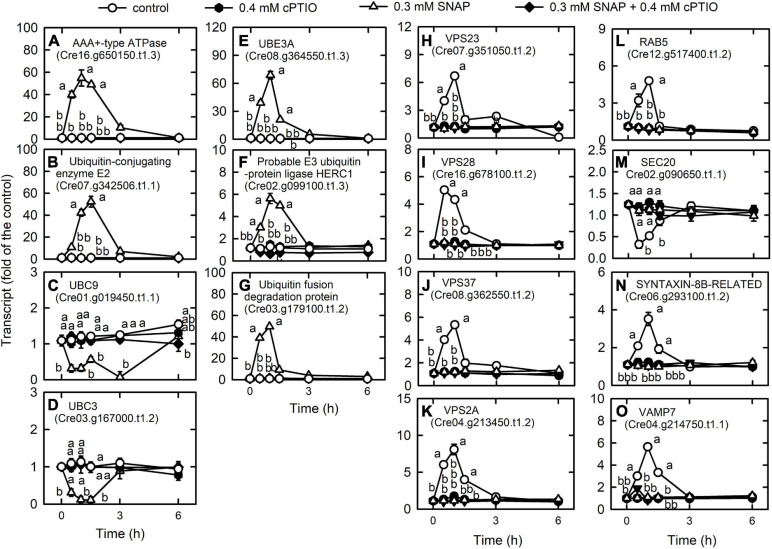
Effects of NO on the expression of genes encoding proteins associated with membrane trafficking system. Time-course changes in the transcript abundances of AAA + -type ATPase **(A)**, ubiquitin-protein ligase E2 **(B)**, UBC9 **(C)**, UBC3 **(D)**, UBE3A **(E)**, probable E3 ubiquitin-protein ligase HERC1 **(F)**, ubiquitin fusion degradation protein **(G)**, VPS23 **(H)**, VPS28 **(I)**, VPS37 **(J)**, VPS2A **(K)**, RAB5 **(L)**, SEC20 **(M)**, SYNTAXIN-8B-RELATED **(N)**, and VAMP7 **(O)** in *C. reinhardtii* after exposure to 0.3 mM SNAP in the presence or absence of 0.4 mM cPTIO. Data are expressed as the mean ± SD (*n* = 3). Different symbols indicate significant differences between treatments (Scheffe’s test, *P* < 0.05).

Based on the identified GO terms, NO induces protein degradation through ubiquitination and membrane trafficking (Supplementary Figure 5). For genes involved in the ubiquitin-proteasome system (Supplementary Table 9), the transcript abundances of AAA + -type ATPase (Cre16.g650150.t1.3), which is responsible for the opening of the gates for substrate into the axial entry ports of the proteases (Yedidi et al., 2017); ubiquitin-protein ligase E2 (Cre07.g342506.t1.2); ubiquitin-protein ligase E3 A (UBE3A; Cre08.g364550.t1.3); probable E3 ubiquitin-protein ligase HERC1 (Cre02.g099100.t1.3); and ubiquitin fusion degradation protein (Cre03.g179100.t1.2), which is similar to ubiquitin fusion degradation protein 1, were increased by NO treatment (Figure 5). In contrast, the transcript abundances of ubiquitin-conjugating enzyme E2I (UBC9, Cre01.g019450.t1.1), which exhibits the activity of small ubiquitin-like modifier (SUMO) E2 conjugase (Wang et al., 2008), a central enzyme in SUMO conjugation for interaction with E1 to accept SUMO in the formation of a SUMO∼UBC9 thioester bond (Pichler et al., 2017), and ubiquitin-conjugating enzyme E2 (UBC3, Cre03.g167000.t1.2) (Figure 5D) decreased in the NO treatment (Figure 5). These changes induced by the SNAP treatment were inhibited in the presence of cPTIO.

**FIGURE 6:**
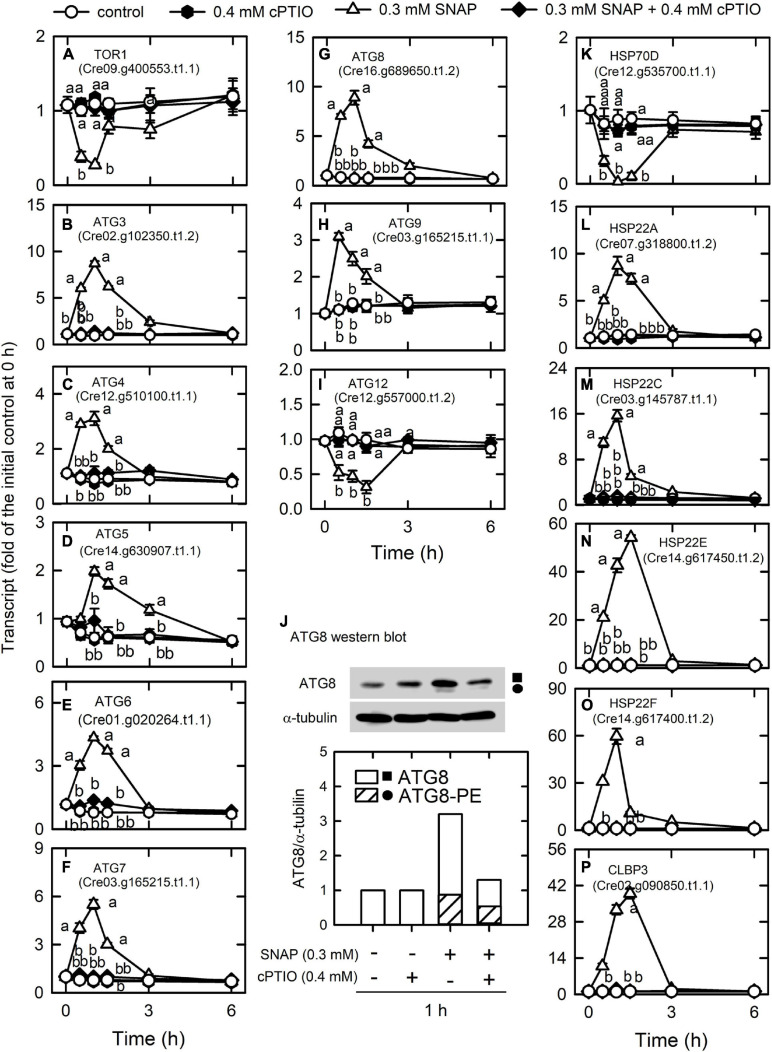
Effects of NO on the expression of genes encoding autophagy proteins. Time-course changes in the transcript abundances of autophagy-related genes and heat-shock proteins and the protein abundances of ATG8 in *C. reinhardtii* upon exposure to 0.3 mM SNAP in the presence or absence of 0.4 mM cPTIO. **(A)** TOR1; **(B)** ATG3; **(C)** ATG4; **(D)** ATG5; **(E)** ATG6; **(F)** ATG7; **(G)** ATG8; **(H)** ATG9; **(I)** ATG12; **(J)** ATG8 (square symbol) and ATG8-PE (circle symbol) protein abundances; **(K)** HSP70D; **(L)** CLBP3; **(M)** HSP22A; **(N)** HSP22C; **(O)** HSP22E; **(P)** HSP22F. Data are expressed as the mean ± SD (*n* = 3). Different symbols indicate significant differences between treatments (Scheffe’s test, *P* < 0.05).

We detected most of the genes encoding endocytosis (Supplementary Table 10A) and SNAREs (Supplementary Table 10B; soluble *N*-ethylmaleimide-sensitive factor attachment protein receptor), which mediate fusion events for autophagosome biogenesis as the membrane trafficking pathways. Nitric oxide increased the transcript abundances of the vacuolar protein sorting (VPS) proteins, which sort receptors within the endocytic pathway (Bishop and Woodman, 2001) including the subunits of the ESCRT-I (endosomal sorting complex I required for transport) complex [i.e., VPS23 (Cre07.g351050.t1.2), VPS28 (Cre16.g678100.t1.2), and VPS37 (Cre08.g362550.t1.2)], the subunit of the ESCRT-III complex (VPS2A; Cre04.g213450.t1.2), and RAB5 (Cre12.g517400.t1.2), a small rab-related GTPase that regulates vesicle formation and membrane fusion. NO also increased the transcript abundances of SYNTAXIN-8B-RELATED (Cre06.g293100.t1.2), a member of the syntaxin family that is involved in protein trafficking from early to late endosomes *via* vesicle fusion (Prekeris et al., 1999), and endosomal R-SNARE protein belonging to the VAMP7 (vesicle associated membrane protein)-like family (R.III) (VAMP7, Cre04.g214750.t1.1), which functions in clathrin-independent vesicular transport and membrane fusion events necessary for protein transport from early endosomes to late endosomes (Advani et al., 1999; Prekeris et al., 1999). Nitric oxide decreased the transcript abundances of endoplasmic reticulum (ER) Qb-SNARE protein and Sec20-family (SEC20, Cre02.g090650.t1.1) (Figure 5). In addition, NO impacted vesicular protein trafficking that is responsible for the accurate delivery of proteins to correct subcellular compartments (Rosquete et al., 2018) *via* tethering of a transport vesicle to its target membrane, which is regulated by two classes of tethering factors, long coiled-coil proteins and multi-subunit tethering complexes (MTCs) (Ravikumar et al., 2017). The transport protein particle (TRAPP) complex is a well-studied MTC in yeast and mammals for the regulation of ER-to-Golgi and Golgi-mediated secretion membrane trafficking (TRAnsport Protein Particle, TRAPPI and TRAPPII) and autophagy (TRAPPIII) processes (Kim et al., 2016; Vukasinovic and Zarsky, 2016). Here, we detected several TRAPP genes in *Chlamydomonas*, in which the transcript abundances of TRAPP I subunits (TRS20, TRS23, TRS31, TRS33, BET3, BET5) and a TRAPPIII subunit (TRS85) were transiently decreased by NO burst (Supplementary Figure 11). As a catabolic process for recycling cellular materials, autophagy is also induced by NO burst (Supplementary Table 10C), which was reflected by a decrease in TOR1 (Cre09.g400553.t1.1) transcript abundance as well as an increase in the transcript abundance of ATG3 (Cre02.g102350.t1.2), ATG4 (Cre12.g510100.t1.1), ATG5 (Cre14.g630907.t1.1), ATG6 (Cre01.g020264.t1.1), ATG7 (Cre03.g165215.t1.1), ATG8 (Cre16.g689650.t1.2), and ATG9 (Cre09.g391500.t1.1) (Figure 6). However, NO decreased the transcript abundance of ATG12 (Cre12.g557000.t1.2) (Figure 6I). ATG8 and ATG8-PE (phosphatidylethanolamine) proteins, which were detected using western blot were also increased by NO burst. The changes in the expression of these genes by SNAP were inhibited in the presence of cPTIO (Figure 6J).

Nitric oxide (NO) also modulates protein folding via the transcriptional control of heat shock proteins (HSPs) that control protein folding and homeostasis in *Chlamydomonas* (Schroda et al., 2015). An overview of how strongly NO treatment impacts expression levels for each HSP gene, including DNAJ-like proteins, small HSPs, HSP60s, HSP70s, HSP90s, and HSP100s, is provided in Supplementary Table 10D. HSP70D (Cre12.g535700.t1.1) was significantly decreased by NO burst and CLBP3 (Cre02.g090850.t1.1, belonging to the HSP100 family) and small HSPs (HSP22A, Cre07.g318800.t1.2; HSP22C, Cre03.g145787.t1.1; HSP22E, Cre14.g617450.t1.2; HSP22F, Cre14.g617400.t1.2) were significantly increased by NO burst (Figure 6). The effect of SNAP on the expression of HSPs was inhibited in the presence of cPTIO.

### Induction of the Antioxidant Defense System by NO

**FIGURE 7:**
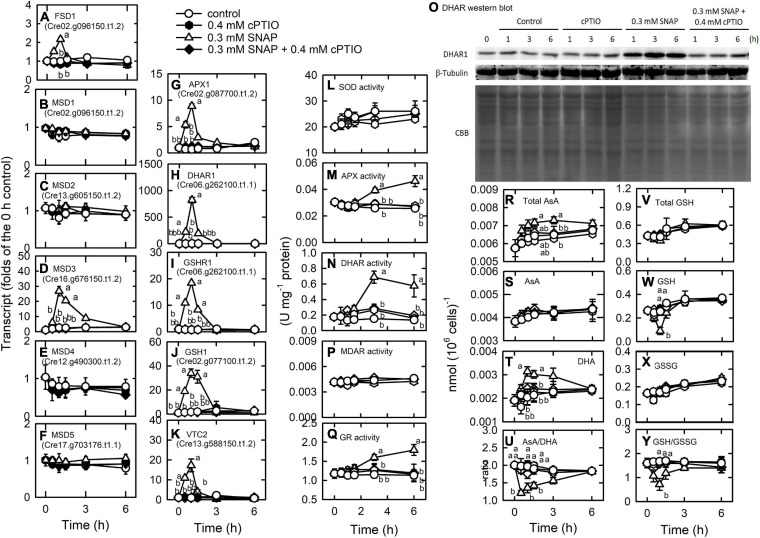
Effects of NO on the expression of genes encoding proteins involved in antioxidant defense system. Time-course changes in the transcript abundances of FSD1 **(A)**, MSD1 **(B)**, MSD2 **(C)**, MSD3 **(D)**, MSD4 **(E)**, MSD5 **(F)**, APX1 **(G)**, DHAR1 **(H)**, GSHR1 **(I)**, GSH1 **(J)**, and VTC2 **(K)**; the activity of SOD **(L)**, APX **(M)**, and DHAR **(N)**, DHAR western blot **(O)**, the MDAR **(P)**, and GR **(Q)** activity; the concentrations of total AsA **(R)**, AsA **(S)**, and DHA **(T)**; the ratio of AsA/DHA **(U)**; the concentrations of total GSH **(V)**, GSH **(W)**, and GSSG **(X)**; and the ratio of GSH/GSSG **(Y)** in *C. reinhardtii* after exposure to 0.3 mM SNAP in the presence or absence of 0.4 mM cPTIO. Data are expressed as the mean ± SD (*n* = 3). Different symbols indicate significant differences between treatments (Scheffe’s test, *P* < 0.05).

Through enzymatic and non-enzymatic routes, the antioxidant defense system is induced by NO treatment (Supplementary Table 11). Superoxide dismutase (SOD), responsible for the dismutaton of O_2_. ^–^ to H_2_O_2_, is among the most important antioxidant defense enzymes in plants (Alscher et al., 2002). Six SODs, FSD1, MSD1, MSD2, MSD3, MSD4, and MSD5, were detected to have the most abundant expression for FSD1 (Supplementary Table 11), in which FSD1 and MSD3 transcript abundances were markedly increased by NO treatment (Figures 7A–F). Furthermore, the ascorbate-glutathione cycle (AGC), an important defense pathway for detoxification of H_2_O_2_, plays a role in the defense of photo-oxidative stress in *Chlamydomonas* (Barth et al., 2014; Chang et al., 2017; Yeh et al., 2019; Kuo et al., 2020b; Lin et al., 2020, 2018) and was also induced by NO. NO increased the transcript abundance of APX1 (Cre02.g087700.t1.2), DHAR1 (Cre10.g456750.t1.2), and GSHR1 (Cre06.g262100.t1.1), but decreased the transcript abundance of APX2 (Cre06.g285150.t1.2), APX4 (Cre05.g233900.t1.2), and MDAR1 (Cre17.g712100.t1.1) (Figure 7 and Supplementary Figure 12). The transcript abundance of GSHR2 (Cre09.g396252.t1.1) was not affected by NO (Supplementary Figure 7). For glutathione (GSH) and ascorbate (AsA) biosynthesis, the transcript abundance of GSH1 (Cre02.g077100.t1.2; Figure 7J) and VTC2 (GDP-L-galactose phosphorylase, Cre13.g588150.t1.2; Figure 7K), a key enzyme for AsA biosynthesis (Urzica et al., 2012), increased under NO treatment, while that of GSH2 (Cre17.g708800.t1.1) was not affected (Supplementary Figure 7E). However, the activity of SOD (Figure 7L), APX (Figure 10M), and MDAR (Figure 7P) was not affected by NO, whereas GR (Figure 7Q), DHAR (Figure 10N), and protein (Figure 7O) did show an increase in activity. These gene expression changes under SNAP treatment were inhibited in the presence of cPTIO (Figure 7).

AsA and GSH homeostasis and their redox states [AsA/DHA and GSH/GSSG (oxidized glutathione)] were affected in the NO treatment. Total AsA (Figure 7R) and DHA (Figure 7T) concentrations increased 1 h after NO treatment and AsA concentration was not affected (Figure 7S). The AsA redox state decreased after 1 h of NO treatment and then recovered (Figure 7U). Total GSH concentration did not change in the NO treatment (Figure 7V), while the GSH concentration decreased (Figure 7W) and the GSSG concentration increased (Figure 7X), which in turn caused a decrease in the GSH redox state that was restored after 3 h (Figure 7Y). These changes were inhibited in the presence of cPTIO.

Furthermore, the transcript abundances of a gene homologous to glutathione peroxidase (GPX5) and σ-class glutathione-*S*-transferase genes (GSTS1, GSTS2), associated with oxidative stress acclimation and detoxification response in *Chlamydomonas* (Ledford et al., 2007; Fischer et al., 2012), also increased under NO treatment. GPX1, GPX, GPX3, and GPX4 showed a decrease in transcript abundance in the NO treatment (Supplementary Figure 13).

Nitric oxide (NO) regulates the expression of methionine sulfoxide reductase (MSR), which functions in the reversibility of the oxidization of methionine to methionine and the control of redox homeostasis through modulating the redox status of methionine in proteins (Branlant, 2012; Rey and Tarrago, 2018). Here, transcript abundances of MSRA3, MSRA5, and MSRB2.2 increased under NO treatment while those of MSRA2, MSRA4, and MSRB2.1 showed a decrease. The transcript abundance of MSRA1was not affected by NO treatment (Supplementary Figure 13). The presence of cPTIO inhibited the changes of MSR gene expression.

**FIGURE 8:**
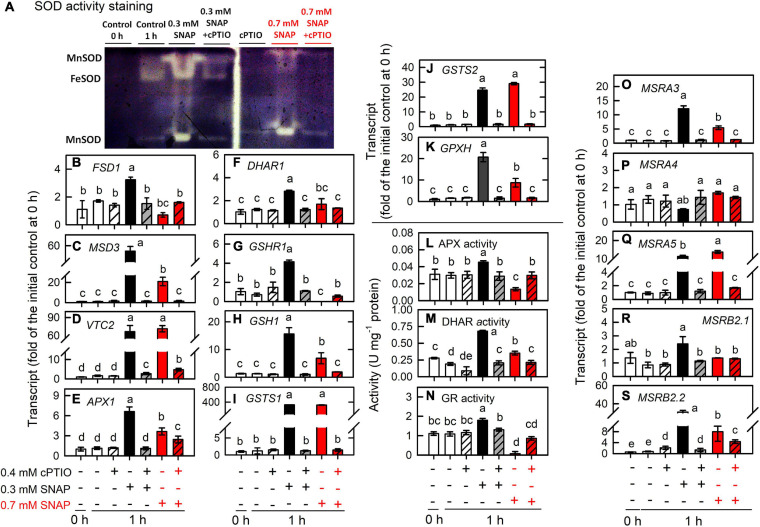
Effects of 0.7 mM SNAP treatment on the expression of genes encoding proteins involved in antioxidant defense system. The transcript abundances of SOD activity assay **(A)**, FSD1 **(B)**, MSD3 **(C)**, VTC2 **(D)**, APX1 **(E)**, DHAR1 **(F)**, GSHR1 **(G)**, GSH1 **(H)**, GSTS1 **(I)**, GSTS2 **(J)**, and GPX5 **(K)**; the activity of APX **(L)**, DHAR **(M)**, and GR **(N)**; the transcript abundance of MSRA3 **(O)**, MSRA4 **(P)**, MSRA5 **(Q)**, MSRB2.1 **(R)**, and MSRB2.2 **(S)** in *C. reinhardtii* 1 h after exposure to 0.7 mM SNAP in the presence or absence of 0.4 mM cPTIO. Data are expressed as the mean ± SD (*n* = 3). Different symbols indicate significant differences between treatments (Scheffe’s test, *P* < 0.05).

Furthermore, the treatment of 0.7 mM SNAP that led to an approximately 50% inhibition in cell growth (cell number) and cell death (SYTOX green fluorescence), which can be prevented in the presence of 0.4 mM cPTIO (Supplementary Figure 14), on the expression of genes associated with antioxidant defense system was assayed. As shown in Figure 8, the increase in the transcript abundances and enzyme activities of SOD (Figures 8A–C), APX (Figures 8E,L), DHAR (Figures 8F,M), and GR (Figures 8G,N) by 0.7 mM SNAP treatment was less than those by 0.3 mM SNAP treatment. The increase in the transcript abundance of VTC2 by 0.7 mM SNAP treatment was similar to that by 0.3 mM SNAP treatment (Figure 8D) while that of GSH1 by 0.7 mM SNAP treatment was less than that by 0.3 mM SNAP treatment (Figure 8H). Similarly, the increase of the transcript abundance of GPXH by 0.3 mM SNAP treatment was also less than that by 0.7 mM SNAP treatment (Figure 8K). Besides, the increase of the transcript abundances of MSRA3 (Figure 8A), MSRB2.1 (Figure 8D), and MSRB2.2 (Figure 8E) by 0.3 mM SNAP treatment was also less than that by 0.7 mM SNAP treatment.

## Discussion

Nitric oxide (NO) is a cellular messenger that mediates diverse signaling pathways and plays a role in many physiological processes in plants (Lamattina and Polacco, 2007; Besson-Bard et al., 2008). Studying NO burst over a short-term period provided us a chance to elucidate the metabolic shift to a brief NO attack and the following acclimation processes in *Chlamydomonas* cells. We recently discovered that NO interacts with reactive oxygen species (ROS) to induce cell death in association with autophagy in *Chlamydomonas* cells under high intensity illumination (Kuo et al., 2020a). Fortunately, ROS over-production and oxidative damage were not found in the 0.3 mM SNAP treatment. Thus, the interference of over-produced ROS in the short-term response to NO can be excluded. Moreover, the cells collected from the mid-exponential phase are not nutrient-starved (remaining ammonium and phosphate concentration in the medium are approximately 41% and 65% of the initial level, respectively). The role of NO as a factor responsible for SNAP-induced changes was confirmed with the cPTIO treatment, which allowed for a better understanding of the novel components of gene networks in *Chlamydomonas* cells against NO stress using transcriptome and physiological analyses. After 1 h of NO treatment, a decrease in the expression of genes associated with transcriptional and translational activity reflected an inhibition of metabolism in *Chlamydomonas* cells under NO stress. However, *Chlamydomonas* growth was slightly impacted by NO stress (0.3 mM SNAP) along with metabolic shifts. The present data suggest that the physiological acclimation of *Chlamydomonas* cells to NO burst can be accomplished by specific metabolic pathways.

Most of the genes with significant expression is modulated during 0.5–1.5 h after SNAP treatment, followed by a recovery after 3 h. It reflects that most metabolism shifts initiate early during the NO burst and resume after 1.5 h. However, some of the genes, including THB1, ARS3, ARS5, UBC9, ATG5, and RBOL2, remains high 3 h after NO treatment. It reflects that NO scavenging system, part of the sulfur starvation response and the protein trafficking system, and NADPH oxidase are still working till 3 h after exposure to NO, which strengthen the execution of acclimation process post NO burst.

**FIGURE 9:**
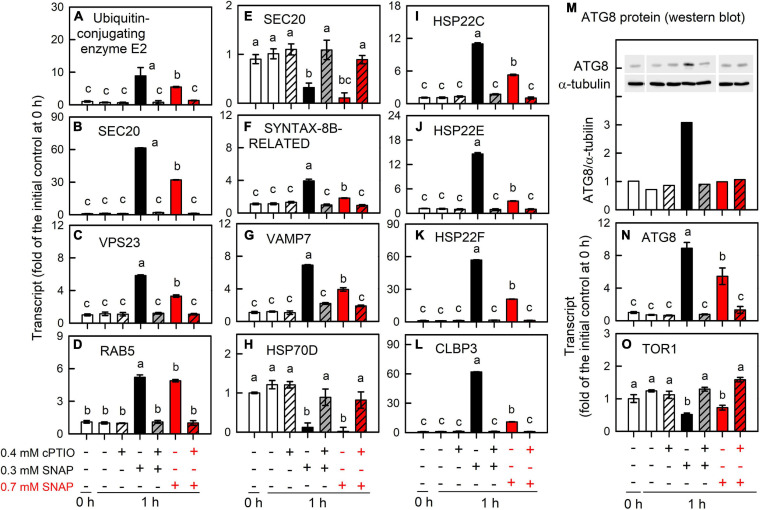
Effects of 0.7 mM SNAP treatment on the expression of genes encoding protein trafficking system. The transcript abundances of A. Ubiquitin-conjugating enzyme E2 **(A)**, UBE3A **(B)**, VPS23 **(C)**, RAB5 **(D)**, SEC20 **(E)**, SYNTAX-8B-RELATED **(F)**, VAMP7 **(G)**, HSP70D **(H)**, HSP22C **(I)**, HSP22E **(J)**, HSP22F **(K)**, CLBP3 **(L)**, and ATG8 **(N)**, and TOR1 **(O)**; the protein abundance of ATG8 **(M)** in *C. reinhardtii* 1 h after exposure to 0.7 mM SNAP in the presence or absence of 0.4 mM cPTIO. Data are expressed as the mean ± SD (*n* = 3). Different symbols indicate significant differences between treatments (Scheffe’s test, *P* < 0.05).

**FIGURE 10:**
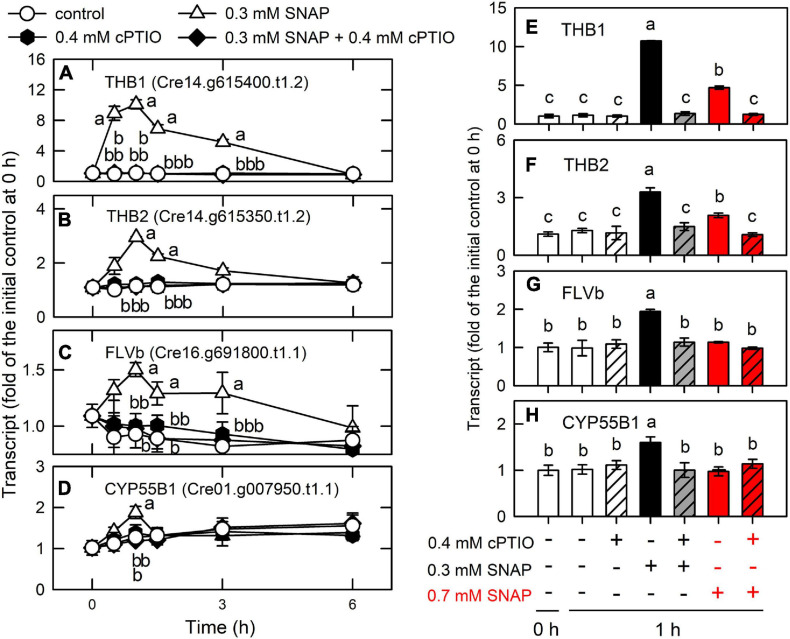
Effects of NO on the expression of genes encoding NO scavenging proteins. Time-course changes in the transcript abundances of THB1 **(A,E)**, THB2 **(B,F)**, FLVB **(C,G)**, and CYP55B1 **(D,H)** in *C. reinhardtii* after exposure to 0.3 or 0.7 mM SNAP in the presence or absence of 0.4 mM cPTIO. The transcript abundances for 0.7 mM SNAP treatment are determined at 1-h samples. Data are expressed as the mean ± SD (*n* = 3). Different symbols indicate significant differences between treatments (Scheffde’s test, *P* < 0.05).

Furthermore, the metabolic underpinnings of *Chlamydomonas* survival under serious NO stress (a partial suppression of long-term cell growth) are compared with the molecular events in response to 0.3 mM SNAP treatment (without growth impairment) for assessing the processes underlying persistent metabolic activity during sustained cell viability. Here, the serious NO stress was established by the treatment with 0.7 mM SNAP, which resulted in an approximately 65% inhibition of cell growth (after 24 h) and viability (SYTOX green staining) and this inhibition can be relieved in the presence of 0.4 mM cPTIO (Supplementary Figure 14). The data obtained from 0.7 mM SNAP treatment (1 h) showed that the induction of NO scavenging system (Figure 10; THB, FLVb, and CYP55B1), antioxidant defense system (SOD, APX, DHAR, GR, GSH1, GPX5, MSRA3, MSRB2.1, and MSRB2.2) (Figure 8), protein trafficking system (VPS, RAB, SNARE, ATG8, and TOR1), and protein chaperone system (small HSPs) (Figure 9) by 0.7 mM SNAP treatment was smaller than 0.3 mM SNAP treatment. The treatment of 0.7 M GSNO showed a similar effect (Supplementary Figure 16). It implicates that NO scavenging activity, antioxidant defense capacity, and protein quality control ability are not sufficiently operating in *Chlamydononas* cells in response to serious NO stress. Whether these processes underlying consistent metabolic activity during sustained cell survival are critical for NO acclimation in *Chlamydomonas* cells needs further experiments.

### Induction of the NO Scavenging System Allows for the Acclimation of *Chlamydomonas* to NO Stress

A transient upregulation of truncated hemoglobin (THB1, Cre14.g615400.t1.2; THB2, Cre14.g615350.t1.2), flavodiiron proteins (FLVB, Cre16.g691800.t1.1), and cytochrome P450 (CYP55B1, Cre01.g007950.t1.1) was found during the period of NO burst (Supplementary Table 7 and Figure 10). In *Chlamydomonas*, THB1 with dioxygenase activity that converts NO into nitrate (Calatrava et al., 2017) is upregulated by NO (Sanz-Luque et al., 2015b). Sanz-Luque et al. (2015a) showed that treatment with 0.1 mM DEANONOate, a NO donor, increased THB1 transcript abundance in the NIT2 (the nitrate assimilation-specific regulatory gene) wild type and the *nit2* mutant, while THB2 transcript abundance decreased in the NIT2 wild type and showed a slight increase in the *nit2* mutant. *Chlamydomonas* is able to reduce NO to N_2_O via FLVB under light conditions (Chaux et al., 2017) or CYP55B1 in the dark (Burlacot et al., 2019). Accordingly, the present data indicate that the NO scavenging system is induced by NO burst to prevent a toxic NO effect, thus allowing the implementation of acclimation processes post NO exposure.

THB1 has been considered as two roles for *Chlamydomonas* cells in dealing NO metabolism in the NO cycle (NO_3_^–^ → NO_2_^–^ → NO → NO_3_^–^): (1). NO scavenging to avoid its toxic effects and (2). regulation of nitrate reduction (Sanz-Luque et al., 2015a; Calatrava et al., 2017). Here, CC125 used in the present study is a *nit1nit2* mutant that cannot utilize nitrate due to the lack of NR, NiR, and HANiT [NIT1 is nitrate reductase (NR) and nit2 is a transcription factor as the nitrate assimilation–specific regulatory gene]. Furthermore, TAP with ammonium as inorganic nitrogen source was used in this study. It is expected that nitrate and nitrile are not existing in *Chlamydomonas* CC125 cells in our current culture system. According to the studies carried out by Prof. Galvan (de Montaigu et al., 2010; Sanz-Luque et al., 2013, 2015a, Chamizo-Ampudia et al., 2016; Calatrava et al., 2017), both ammonium and NO are the negative signals that affect the expression of the genes involved in nitrate assimilation transcriptionally and post-transcriptionally. The nitrate/nitrile transporters at the plasma and chloroplast membranes are under the control of the regulator NIT2. Therefore, the CC125 strain does not express the proteins associated with nitrate/nitrile transporters. It is obvious that nitrate assimilation does not occur in *Chlamydomonas* strain CC125. Although NO can be converted to nitrate through NR/THB1 partner in the NO cycle, NO released from SNAP or GSNO cannot be converted to nitrate via THB1 due to the deficiency of NR in strain CC125, even THB1 is markedly upregulated by short-term NO burst in the present study.

### A Shutdown of Transcriptional and Translational Activity and Nitrogen/Sulfur Assimilation and an Alteration in Primary Amino Acid Biosynthesis by NO Burst

Current results that the genes associated with transcriptional and translational regulation are the majority of downregulated genes by NO burst (Supplementary Tables 5, 6) suggest that protein synthesis is significantly attenuated after NO treatment.

In addition to the blockage of protein synthesis, the availability of nitrogen as well as sulfur for amino acid synthesis is restricted by sudden NO challenge, as reflected by a transient drop in the expression of ammonium transporters and assimilation enzymes, whereas an increased SAC3 expression was observed, which is related to the inhibition of the expression of most ARSs. Moreover, the temporal upregulation of genes involved in the degradation of several amino acids, including sulfur-containing amino acids, under NO stress suggests that NO burst decreased the synthesis of proteins and other nitrogen-containing compounds that use these amino acids as building blocks. NO also causes a reversible inhibition of high-affinity nitrate/nitrite and ammonium transport and NR activity through post-translational regulation (Sanz-Luque et al., 2013). However, time-course changes in transcription levels reveal that the impedance in nitrogen/amino acid and sulfur utilization by NO burst is relieved after 3 h. The upregulation of sulfate transporters (SLT1, SLT2, and SULP3) and GDH1 functioning in the incorporation of ammonium in *Chlamydomonas* (Muñoz-Blanco and Cárdenas, 1989) serves a purpose for improving sulfur availability and amino acid synthesis under NO stress. However, the process by which nitrogen/sulfur assimilation is limited under NO stress is relieved post 3 h.

### Mechanisms to Overcome NO-Induced Protein Stress

A decrease in the expression of the genes encoding the oligosaccharyltransferase complex, including RNP1 [oligosaccharyltransferase complex subunit delta (ribophorin II)], GTR17 (Glycosyltransferase), GTR22, GTR25 (oligsaccharyltransferase STT3 subunit), STT3B (dolichyl-diphosphooligosaccharide-protein glycosyltransferase subunit STT3B), CANX [calnexin (CANX)], and CRT2 (Calreticulin 2, calcium-binding protein), by NO treatment (Supplementary Table 7) reflects the dysfunction of proteins caused by a blockage of glycosylation in the ER. However, the expression of DAD1, which functions in N-linked glycosylation in the ER (Kelleher and Gilmore, 1997), increased in the NO treatment. The NO-induced downregulation of TRAPPI and III proteins, which are related to the transport of proteins to correct compartments and the formation of autophagosomes (Rosquete et al., 2019; Garcia et al., 2020), also suggests that there was transient transfer of misfolded and damage proteins during NO treatment. Furthermore, the downregulation of SUMO E2 conjugase, UBC9, by NO reflects the inhibition of SUMOylation for post-translational modification of the proteins, which is crucial for the growth of *Chlamydomonas* cells (Knobbe et al., 2015). Clearly, NO burst causes protein stress in *Chlamydomonas*.

To overcome disorders caused by potentially misfolded proteins, the membrane trafficking system is induced for the degradation of damaged proteins in the lysosome and/or vacuole via autophagy, which was reflected by a transient increase in the expression of E2 and E3 ubiquitin ligases, ESCRT subunits (VSP), SNAREs, and ATGs by NO treatment. The increase in the expression of SYNTAXIN-8B-RELATED and VAMP7, which are endosomal syntaxins that mediate the steps of endosomal protein trafficking (Prekeris et al., 1999), in *Chlamydomonas* cells suggests that there was fusion between autophagosomes (late endosomes) and lysosomes under NO stress. The TEM images show the formation and fusion of large vesicles at 1 h, possibly acting to enclose the cytosolic components for degradation (Xie and Klionsky, 2007; Nakatogawa et al., 2009); the large vesicles were not visible after 6 h (Supplementary Figure 15). Using autophagy as a lysosome-mediated pathway for the degradation of cytosolic proteins and organelles (Choi et al., 2013), ATG7 is involved in the two ubiquitin-like systems necessary for the selective cytoplasm-to-vacuole targeting (Cvt) pathway and autophagy by activation of ATG12 and ATG8 and assigns them to E2 enzymes, ATG10 and ATG3, respectively. During this process, ATG8 conjugates PE to form lipidated ATG8 (ATG8-PE), an autophagosome membrane component and binding partner for autophagy receptors, which act to recruit cargo to lysosomes for catabolism in ubiquitin-dependent and independent manners for selective autophagy. Although ATG12 and VTC1 (vacuolar transport chaperone-like protein, Cre12.g510250 t1.2) were downregulated by NO burst, an increase in the expression of other ATG genes and abundances of ATG/ATG8-PE proteins in NO-treated *Chlamydomonas* cells demonstrates the induction of autophagy by working with the sorting protein factors involved in the membrane trafficking system for protein degradation and recycling under NO stress. Because the inhibition of TOR activity can trigger autophagy, as evidenced by the increased ATG8 and ATG8-PE proteins in *Chlamydomonas* (Pérez-Pérez et al., 2010), the decreased expression of TOR1 due to NO treatment indicates that TOR plays a role in autophagy induction. In addition to membrane trafficking, protein chaperone systems (HSP22A, HSP22C, HSP22E, HSP22F, and CLBP6) are induced in *Chlamydomonas* cells under NO stress. In response to sub-lethal NO treatment (0.7 mM SNAP), a smaller increase in the transcript abundances of ubiquitin-conjugating enzyme E2, UBE3A, VPS23, RAB5, SYNTAX-8B-RELATED, VAMP7, HSP22C, HSP22E, HSP22F, CLBP3, and ATG8 than that by 0.3 mM SNAP treatment (Supplementary Figure 16) suggests a role of protein trafficking system for NO acclimation. Thus, the protein trafficking system (ubiquitination, SNARE, autophagy) and HSPs are evoked as a way to remove aberrantly folded or damaged proteins, allowing *Chlamydomonas* cells to maintain normal functions under NO stress.

### NO Inhibits Photosynthesis but Induces the Antioxidant Defense System for the Prevention of Oxidative Stress Upon Exposure to NO Burst

Our results agree the NO-mediated shutdown of photosynthesis in *Chlamydomonas* cells inder nitrogen starvation condition (Wei et al., 2014). A decrease in the expression of genes encoding proteins related to photosynthesis and photosynthetic activity in *Chlamydomonas* by NO illustrates the NO-mediated downregulation of PSII activity and the evolutionary rate of photosynthetic O_2_ at the transcriptional level. Because of the suppression of PSII activity and the photosynthetic O_2_ evolution rate by the NO treatment at the protein level in higher plants (Takahashi and Yamasaki, 2002; Singh et al., 2007; Wodala et al., 2008; Ördög et al., 2013), the effects of NO on photosynthesis by protein modification cannot be ignored. NO is also a factor leading to the degradation of the cytochrome *b*_6_*f* complex and Rubisco through FtsH and Clp proteases in sulfur (de Mia et al., 2019) or nitrogen (Wei et al., 2014) starved *Chlamydomonas* cells. However, it is not clear whether these two protein complexes are degraded under NO burst in nutrient sufficient conditions. We did find that the transcript abundances of the FtsH-like proteases FHL4 (Cre13.g568400.t1.2) and FHL6 (Cre03.g201100.t1.2), and DEG11 (Cre12.g498500.t1.2) were increased by NO burst, whereas the transcript abundances of the ClpP proteases ClpP4 (Cre12.g500950.t1.2) and ClpP5 (Cre12.g486100.t1.2), and the non-catalytic subunit of the ClpP complex, ClpR2 (Cre16.g682900.t1.2) decreased (Supplementary Table 7). However, the role of FtsH-like and DEG proteases responsible for chloroplast protein degradation by NO burst need to be identified. Because the inhibition of photosynthesis is considered the mechanism for algae to acclimate to nitrogen or sulfur limitation to avoid photo-damage (Peltier and Schmidt, 1991; Grossman, 2000; Salomon et al., 2013), our findings suggest that the transient inhibition of photosynthesis can prevent *Chlamydomonas* cells from over-producing ROS, which may occur during NO burst. ROS scavenging ability was enhanced with the upregulation of SOD (FSD1 and MSD3) and APX expression (transcript abundance at 1 h and enzyme activity during 3–6 h), the increased concentration of AsA due to the upregulation of VTC2 and its regeneration rate, owing to the enhanced DHAR expression (transcript abundance at 1 h and protein abundance and enzyme activity during 3–6 h). In addition, the enhanced GSH regeneration rate, supported by the increase in the GR activity (3–6 h) and GSHR1 expression as well as increased GSTS1, GSTS2, and GPX5 expression, prevented the over-production of ROS. Tocopherols (Havaur et al., 2005; Krieger-Liszkay and Trebst, 2006) and carotenoids (Ramel et al., 2012) are also ^1^O_2_ quenchers, but their concentrations and the transcript abundances of their biosynthetic enzymes decreased in the NO treatment (Supplementary Figure 17). Instead, an increase in the concentration of AsA, an ^1^O_2_ quencher (Kramarenko et al., 2006), was responsible for ^1^O_2_ scavenging under NO stress. A transient increase of APX, DHAR, and GR transcript abundances at 1 h, followed by a continuous increase in APX, DHAR, and GR activities during 3–6 h, indicates that the increase in the activities of these AGC enzymes is at least due to transcriptional regulation. It may be due to a post-transcriptional and/or translational regulation of these gene expression. A significant induction of the AGC cycle during 3–6 h is responsible for scavenging of ROS potentially generated during the long-term NO stress. The results of Figure 8 and Supplementary Figure 18 support the necessity of AGC in *Chlamydomonas* cells against NO stress. Therefore, ROS are not over-accumulated post NO burst, allowing the acclimation of *Chlamydomonas* cells when exposed to NO stress.

Nitric oxide (NO) also triggers MSR expression. Although the redox of AsA and GSH as well as the thiol-based redox regulation proteins, TRX2 (thioredoxin-like protein, Cre03.g157800 t1.1), PRX1 (2-cys peroxiredoxin, chloroplastic, Cre06.g257601 t1.2), and PRX2 (2-cys peroxiredoxin, Cre02.g114600 t1.2; Supplementary Table 7), were initially decreased by NO burst, they recovered 3 h after treatment (the restoration of TRX2, PRX1, and PRX2 transcript abundance is not shown). Thus, the antioxidant defense system and MSR worked together to prevent oxidative stress and the abrupt loss in cellular redox homeostasis occurring under NO stress.

### A Differential Regulation of NADPH Oxidase Genes by NO

**FIGURE 11:**
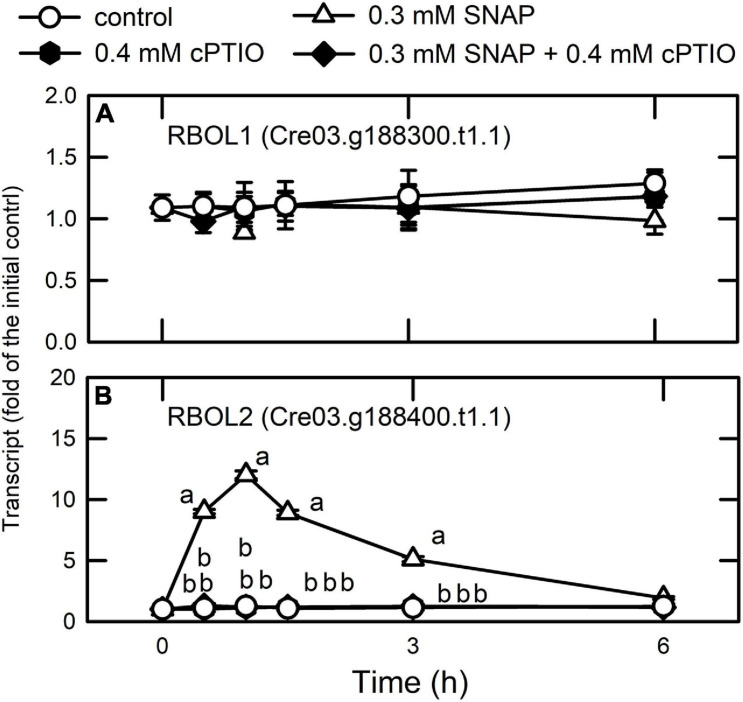
Effects of NO on the expression of genes encoding NADPH oxidase. Time-course changes in the transcript abundances of RBOL1 **(A)** and RBOL2 **(B)** in *C. reinhardtii* after exposure to 0.3 mM SNAP in the presence or absence of 0.4 mM cPTIO. Data are expressed as the mean ± SD (*n* = 3). Different symbols indicate significant differences between treatments (Scheffe’s test, *P* < 0.05).

NADPH oxidase acts as an important molecular hub during ROS-mediated signaling in plants (Marino et al., 2011) and in the interplay of ROS and NO signaling pathways for the regulation of plant metabolism (Suzuki et al., 2011). A previous study has shown that NADPH oxidase is regulated by transcriptional and enzyme activity levels in higher plants (Hu et al., 2020). The NO-mediated suppression of NADPH oxidase activity is due to post-translational modification known as *S*-nitrosylation (Wang et al., 2013). The analysis of *cis*-regulatory elements in the gene promoter region of NADPH oxidase showed diverse expression patterns under different conditions (Wang et al., 2013; Kaur and Pati, 2016). In this study, we did not examine whether the *S*-nitrosylation of NADPH oxidase occurs in *Chlamydomonas* upon exposure to NO; however, our present data did detect the constant expression of the gene encoding respiratory burst oxidase-like 1 (RBOL1, Cre03.g188300.t1.1), an NADPH oxidase gene (Figure 11A). In addition, we observed the significant upregulation of RBOL2 (Cre03.g188400.t1.1) (Figure 11B) in *Chlamydomonas* after 1 h of NO exposure, followed by the subsequent restoration to a baseline level after 3 h.

Whether NADPH oxidase (RBOL2)-dependent molecular events underlie the acclimation mechanisms in *Chlamydomonas* related to coping with NO stress is now undertaken. Furthermore, the direct targets of NO and the mechanisms for the transcriptional regulation of RBOL2 in *Chlamydomonas* are needed to be clarified in the future.

## Conclusion

**FIGURE 12:**
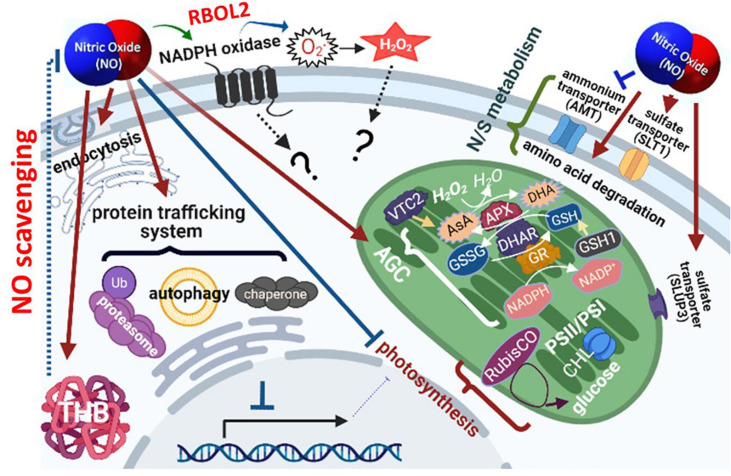
Schematic model of nitric oxide (NO) responsive molecular events in *Chlamydomonas reinhardtii*. The orchestrated implementation of metabolic processes in response to NO is achieved by 1. the modulation of NADPH oxidase (RBOL2) and/or ROS signaling pathway for the regulation of downstream metabolisms, 2. reduction of NO amount via scavenging elements, 3. transient inhibition of photosynthesis together with the induction of the antioxidant defense system and modification of the redox state to prevent oxidative stress, 4. transient attenuation of transcriptional and translational capacity accompanying with the upregulation of the protein trafficking system (ubiquitin, SNARE, and autophagy) and molecular chaperone system for dynamic regulation of protein homeostasis and function, and 5. enhancement of amino acid catabolism and nitrogen/sulfur availability.

The transcriptome analysis, qPCR assay, and physiological changes in the present study, the acclimation of *Chlamydomonas* to NO stress comprises a temporally orchestrated implementation of metabolic processes including 1. modulation of NADPH oxidase (RBOL2) and ROS signaling pathways for downstream mechanism regulation, 2. reduction of NO amount via scavenging elements, 3. transient inhibition of photosynthesis together with the induction of the antioxidant defense system and modification of the redox state to prevent oxidative stress, 4. transient attenuation of transcriptional and translational capacity accompanying with the upregulation of the protein trafficking system (ubiquitin, SNARE, and autophagy) and molecular chaperone system for dynamic regulation of protein homeostasis and function, and 5. enhancement of amino acid catabolism and nitrogen/sulfur availability (Figure 12). The role of NADPH oxidase in the modulation of molecular events underlie the acclimation mechanisms in *Chlamydomonas* related to coping with NO stress is needed to be clarified in the near future.

## Data Availability Statement

The original contributions presented in the study are publicly available. This data can be found here: The transcriptome sequences can be accessed from the Sequence Read Archive (SRA) website using the BioProject accession number: PRJNA629395 and the BioSample accessions SAMN14775313, SAMN14775314, SAMN14775315, SAMN14775316, SAMN14775317, SAMN14775318, SAMN14775319, SAMN14775320, SAMN14775321, and SAMN14775322. Other relevant data are included in the article/Supplementary Material.

## Author Contributions

EYK performed the physiological analysis, RNA extraction, cDNA preparation, and qPCR. TML conceived and designed the experiments, interpreted the data, and wrote the manuscript. All authors contributed to the article and approved the submitted version.

## Conflict of Interest

The authors declare that the research was conducted in the absence of any commercial or financial relationships that could be construed as a potential conflict of interest.

## Publisher’s Note

All claims expressed in this article are solely those of the authors and do not necessarily represent those of their affiliated organizations, or those of the publisher, the editors and the reviewers. Any product that may be evaluated in this article, or claim that may be made by its manufacturer, is not guaranteed or endorsed by the publisher.
