# Supplementary material for: Molecular Mechanisms Underlying the Acclimation of Chlamydomonas reinhardtii Against Nitric Oxide Stress
Source: Front Plant Sci. 2021 Aug 5;12:690763. doi: 10.3389/fpls.2021.690763 (PMC8374494; doi:10.3389/fpls.2021.690763)
Supplement: Supplementary file 1 [file Data_Sheet_1.PDF]

Supplementary Figure

Molecular mechanisms underlying the acclimation of *Chlamydomonas reinhardtii* against nitric oxide stress

Eva YuHua Kuo and Tse-Min Lee

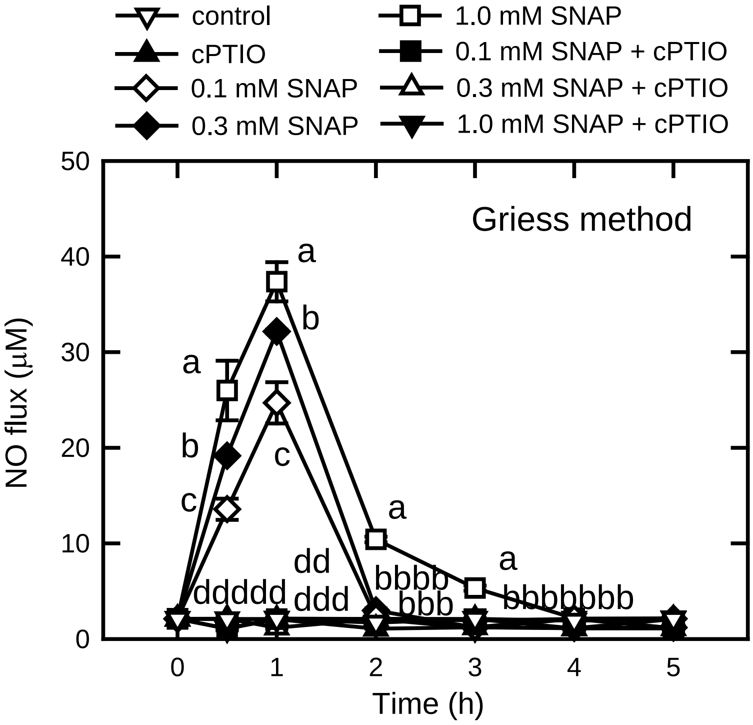

Supplementary Figure S1. **Time-course changes in the NO flux rate in *Chlamydomonas reinhardtii* cells upon exposure to 0.1, 0.3. or 1.0 mM SNAP treatments in the presence or absence of 0.4 mM cPTIO.** Data are expressed as the mean ± SD (n = 3). Different symbols indicate significant differences between treatments at the same time (Scheffe test, $P<0.05$).

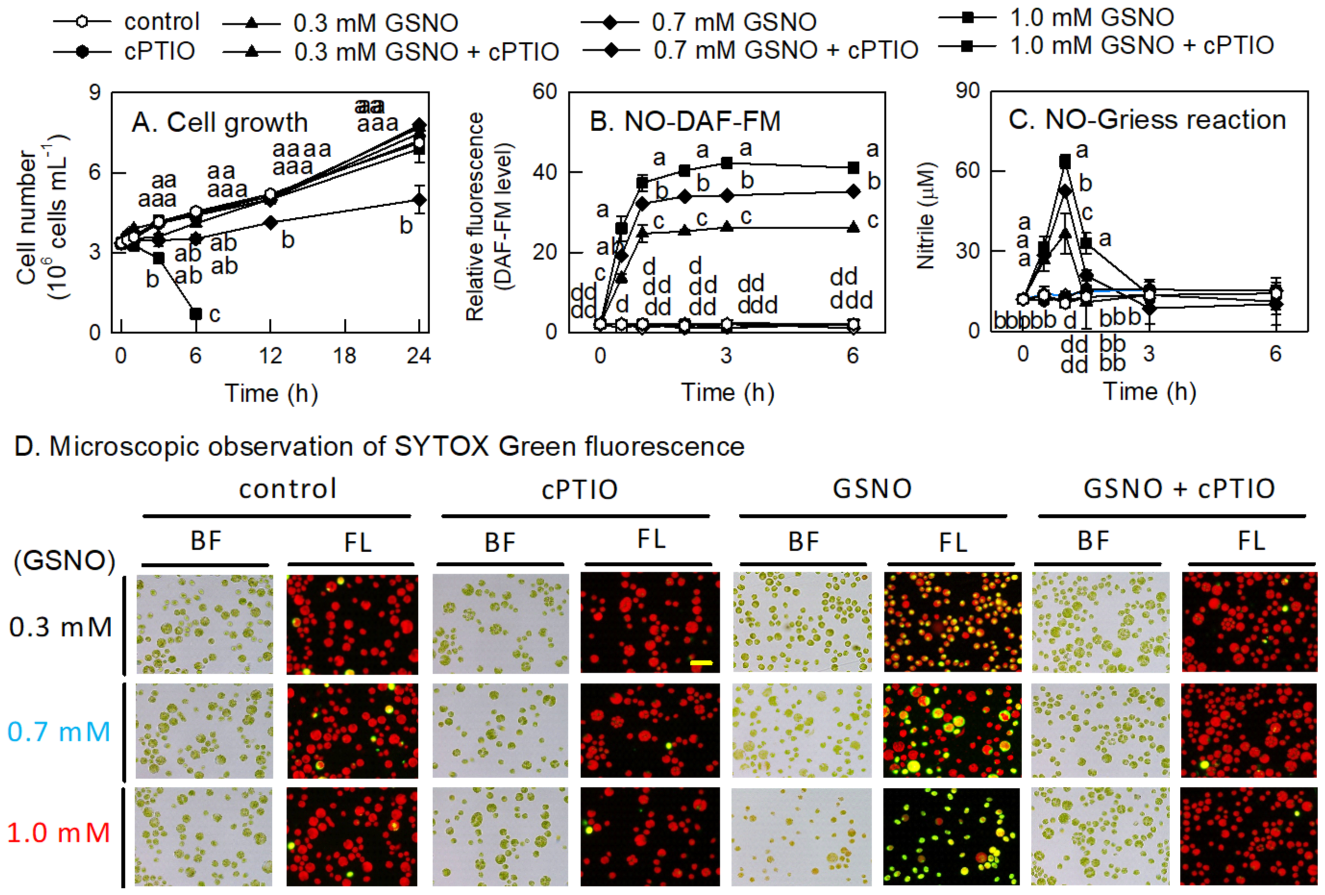

Supplementary Figure S2. Physiological response and the production of NO in *Chlamydomonas reinhardtii* in response to 0.3, 0.7, or 1.0 mM SNAP in the presence or absence of 0.4 mM cPTIO. A, Cell growth; B, DAF-FM fluorescence; C, NO production by Griess reaction; D, Cell death assessed by SYTOX green fluorescence. Data are expressed as the mean ± SD (n = 3). Different symbols indicate significant differences between treatments (Scheffe's test, $P < 0.05$). Bar in photo indicates 25 μm.

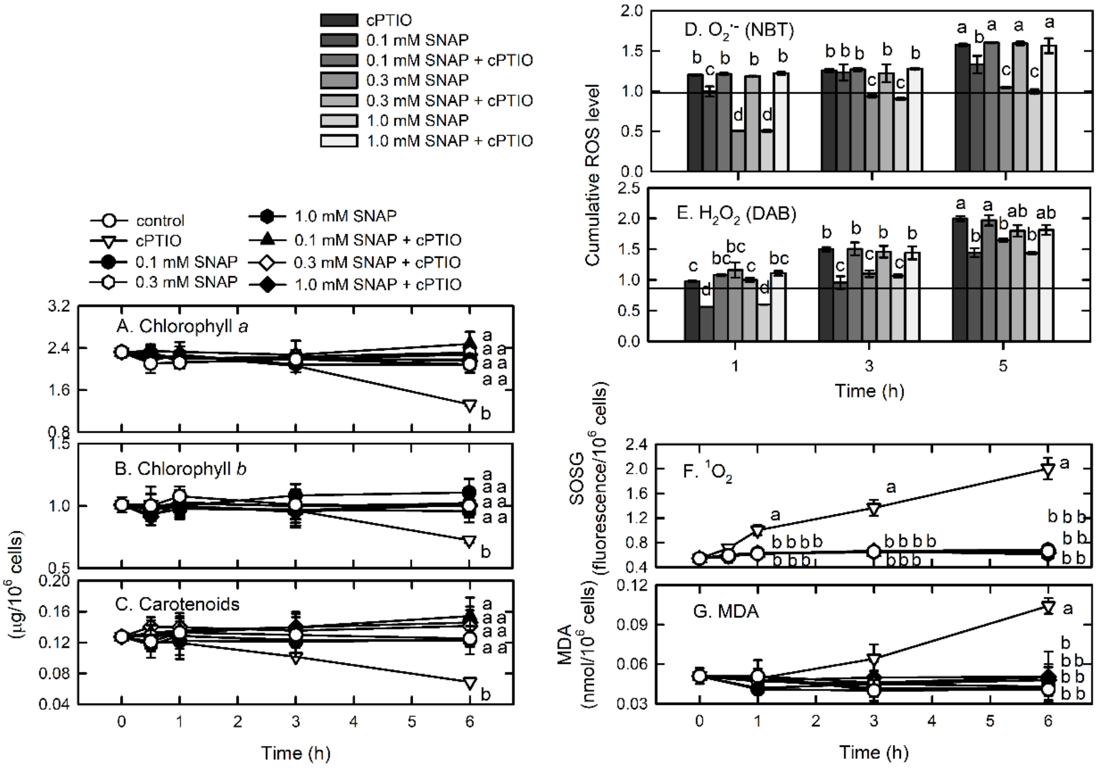

Supplementary Figure S3. Time-course changes in the concentrations of chlorophyll *a* (A), chlorophyll *b* (B), and carotenoids (C), and superoxide anion radical ($O_2^{\cdot -}$) (D), hydrogen peroxide (H_2O_2) (E), and singlet oxygen ($^1O_2$) (F) concentrations in *Chlamydomonas reinhardtii* cells upon exposure to 0.1, 0.3. or 1.0 mM SNAP treatments in the presence or absence of 0.4 mM cPTIO. Data are expressed as the mean ± SD (n = 3). Different symbols indicate significant differences between treatments at the same time (Scheffe test, P<0.05).

Materials and Methods

Determination of Carotenoid Contents

A 1 mL aliquot of the cell suspension for each sample was centrifuged, and the pellet was re-suspended in 1 mL of 80% acetone. After incubation overnight at 4°C in the dark, the acetone extract was centrifuged at 12,000 xg for 5 min at room temperature, and the absorbance of the supernatant was detected at 480, 645, and 663 nm using a Hitachi spectrophotometer to estimate carotenoid concentration using the following equations: total carotenoid (mg mL^{-1}) = A_{480}+ 0.114 (A_{663}) – 0.638 (A_{645}).

Determination of H_2O_2, $O_2^{\bullet -}$, and $^1O_2$ contents

H_2O_2 and $O_2^{\bullet -}$ were detected using 3,3’-diaminobenzidine (DAB)-HCl (Sigma, St. Louis, MO) and nitroblue tetrazolium (NBT; Sigma) (Hema et al. 2007), respectively. The cells were pretreated with NBT and DAB before high-intensity illumination. The cells were centrifuged at 3000g (Centrifuge 5810R, Eppendorf AG) using a swing-

bucket rotor (F-34-6-38, Eppendorf AG) for 3 min at room temperature. The pellet was re-suspended in new TAP medium containing 0.5 mM NBT or 5 mM DAB for a 10-min incubation in the dark. Subsequently, NO donors or cPTIO were added in the TAP medium. At each time point, the cells were filtered onto glass microfiber filters (diameter 45 mm, GF/C, Whatman, GE Healthcare, Piscataway, NJ). The pigments were completely removed following the wash of the filter discs twice with methanol. After drying, the filters were scanned as digital images and staining intensities were estimated using IMAGEJ software (free software from http://rsbweb.nih.gov/ij/index.html) and compared between treatments. To confirm the detection of $O_2^{\bullet-}$ and H_2O_2 by NBT and DAB, 100 U mL^{-1} bovine erythrocytes superoxide dismutase (SOD) (Sigma) was added together with NBT and 100 U mL^{-1} bovine liver catalase (Sigma-Aldrich) was also added together with DAB. SOD and catalase could effectively reduce the blue color and the brown color in the algal cells, respectively. This indicates that the color development after DAB and NBT staining was mainly due to H_2O_2 and $O_2^{\bullet-}$, respectively.

The production of $^1O_2$ was detected using the SOSG dye (Molecular Probes, Invitrogen Inc.). After chemical treatment, the SOSG dye was added to 1 mL of cell culture (in a 2 mL Pyrex test tube, 1.4 cm internal diameter) to achieve a final concentration of 5 mM, followed by a further incubation under LL or VHL condition for 5 min, and then transferred to a 1.5 ml microcentrifuge tube. After washing with TAP medium following centrifugation, the pellet was suspended in 1 mL of fresh TAP medium, and fluorescence was detected at 525 nm (excitation 488 nm) by a fluorescence spectrophotometer (F-2500, Hitachi). Fluorescence was also observed under fluorescence microscopy (Eclipase Ni, Nikon) with excitation at 488 nm using an FITC filter (Nikon). Because SOSG is sensitive to light, staining of $^1O_2$ in the cells using the SOSG dye was carried out in the dark. An SOSG blank was also produced by the addition of SOSG to TAP medium and incubation in the dark for 10 min. We observed that the fluorescence in the SOSG blank was low (data not shown), indicating that the emission of SOSG fluorescence was not due to the light-induced fluorescence of SOSG. Additionally, the SOSG green fluorescence could be markedly reduced in the presence of 15 mM DPA, an $1O_2$ quencher, and enhanced in the medium containing 50% D_2O, and the fluorescence could not be detected when SOSG dye was added in the cells incubated in the dark (data not shown). These results suggest that $^1O_2$ can be detected using SOSG.

Thiobarbituric Acid Reactive Substance (TBARS) Assay

The TBARS assay (MDA) was used to estimate the peroxidation of lipids in membranes or biological systems (Health and Packer 1968). Five milliliters of algal

culture was sampled after treatment and centrifuged at 4,000 xg for 5 min. The pellet was fixed in liquid nitrogen and mixed vigorously with 0.5 mL of 5% (w/v) trichloroacetic acid (TCA). The mixture was subjected to three frozen (-80°C)-thaw (25°C) cycles and centrifuged at 12,000 x*g* for 10 min at 4°C. The supernatant was collected for the determination of lipid peroxidation, and the extent of lipid eroxidation was estimated from the TBARS content determined according to Health and Packer (1968). The TBARS content was calculated based on the A_{532}–A_{600} with an extinction coefficient of 155 mM^{-1} cm^{-1} and expressed as pmol (10^{6} cells)$^{-1}$.

Literature Cited

Heath, R. L. and Packer, L. (1968). Photoperoxidation in isolated chloroplasts. I. Kinetics and stoichiometry of fatty acid peroxidation. *Arch. Biochem. Biophys.* 125, 180-198.

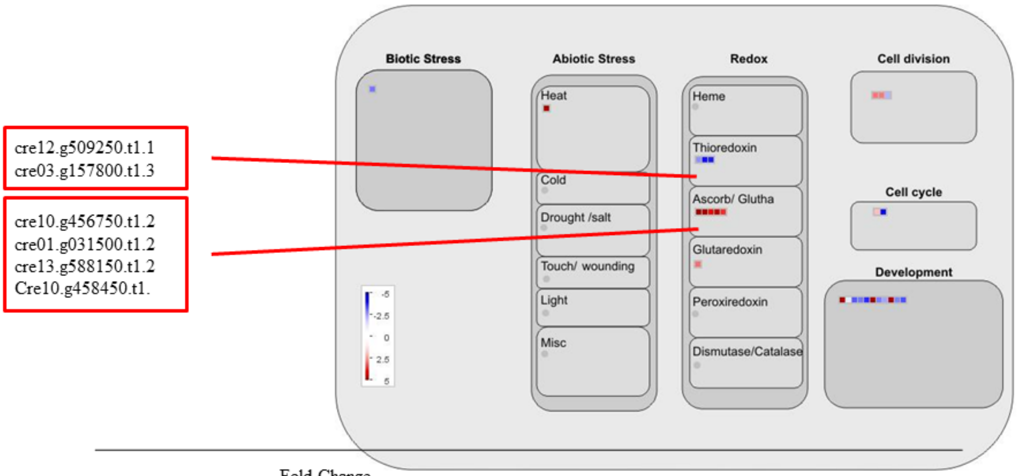

Gene	Fold Change (Log_2)	*P*-value	Seq. description
Cre12.g509250.t1.1	-2.66	0.017	thioredoxin superfamily protein required for eyespot assembly
Cre03.g157800.t1.1	-3.89	0.036	thioredoxin-like protein
Cre13.g588150.t1.2	4.84	0.031	GDP-L-galactose phosphorylase (VTC2)
Cre10.g456750.t1.2	6.73	0.008	dehydroascorbate reductase
Cre01.g031500.t1.2	4.07	0.000	L-ascorbate oxidase
Cre10.g458450.t1.3	3.69	0.026	glutathione peroxidase

Supplementary Figure S4. The genes belonging to cellular response overview according to the MapMan analysis of *Chlamydomonas reinhardtii* cells in response to 0.3 mM SNAP treatment for 1 h as compared to the control.

The next generation sequencing (NGS) using the Illumina technology and de novo assembly by Trinity (version r2012-10-05) (Grabherr et al., 2011) showed that the total length of the illumina reads was > 7.75 gigabases (Gb), equivalent to the ~69-fold coverage of a genome of *C. reinahrdtii* size (112.3 megabases (MB), *Chlamydomonas reinahrdtii* v 5.5) (Merchant et al., 2007) and the average contig size exceeded 415 bps in all 4 libraries with coverage from 87.21-92.21% (Supplemental Table S2). Among the DEGs (Supplemental Table S3), a total of 1,005 significant DEGs was identified based on the critieria of 1.2 log_2FC and *P*-value of $log_2FC \leq 0.05$ (463 upregulated genes and 542 downregulated genes) (Supplemental Table S4). First, the MapMan analysis of these regulated genes shows that cellular response overview (this Figure), proteasome and autophagy (see below, Supplemental Fig. S3), photosynthesis (electron transport, Calvin cycle, and photorespiration) (see below, Supplemental Fig. S4), and tetrapyrrole pathway (see below, Supplemental Fig. S5) were affected under NO stress. Then, the analysis of all the functional DEGs and unigenes through Blast2GO suite shows there are 45 GO terms (score ≤ 0.05) can be assigned to 463 genes (46.07 %) based on gene homology with 13 GO terms to biological process, 30 to molecular function, and 2 to cellular component for upregulated genes (see below, Supplementary Fig. S6A; Supplementary Table S5A), while 157 GO terms for 542 downregulated genes with 337 known function genes (53.93%) are classified into 35 GO terms belong to biological process, 112 to molecular function, and 8 to cellular component (see below, Supplementary Figure S6B; Supplementary Table S5B).

To obtain the extensively insight of transcriptome regulated by NO, the expression level greater than 2 fold-change (1,005 significantly different expression genes with

up- and down-regulated, $P < 0.05$) were selected for MapMan analysis. MapMan allows the visualization in diagrams of gene expression changes of regulated genes, with genes grouped in “BINs” by function or class (Thimm et al., 2004). In the resulting MapMan diagram for *C. reinhardtii* metabolism, displaying alterations in gene expression between the control and NO (0.3 mM SNAP) treatment.

Literature Cited

Grabherr, M. G., Haas, B. J., Yassour, M., Levin, J. Z., Thompson, D. A., Amit, I., Adiconis, X., Fan, L., Raychowdhury, R., Zeng, Q., et al. (2011). Trinity: reconstructing a full-length transcriptome without a genome from RNA-Seq data. *Nat. Biotechnol*. 29, 644-652

Thimm, O., Bläsing, O., Gibon, Y., Nagel, A., Meyer, S., Krüger, P., Selbig, J., Müller, L. A., Rhee, S. Y. and Stitt, M. (2004). MAPMAN: a user-driven tool to display genomics data sets onto diagrams of metabolic pathways and other biological processes. *Plant J.* 37, 914-939.

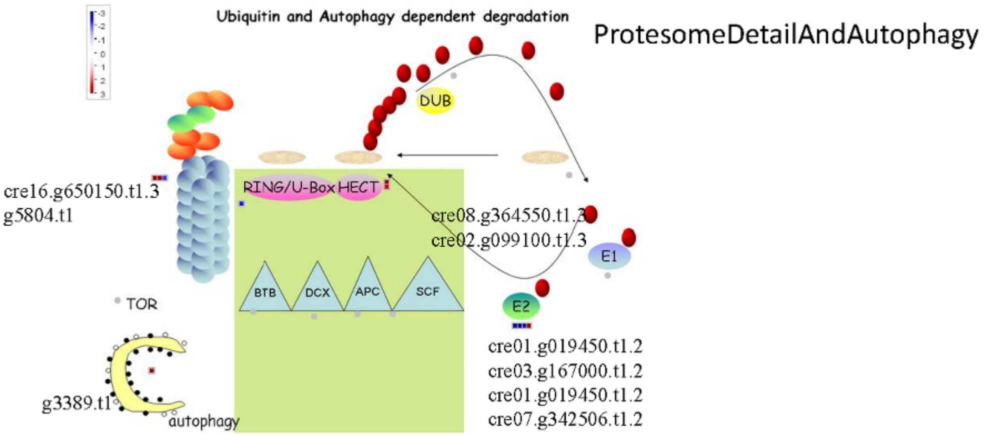

Gene	Fold Change (Log$_2$)	*P*-value	Seq. description
cre16.g650150.t1.3	3.61	0.048	AAA+-type ATPase
cre08.g364550.t1.3	`4.70	0.003	Ubiquitin-protein ligase E3 A
cre02.g099100.t1.1	2.60	0.005	Probable E3 ubiquitin-protein ligase HERC1
cre01.g019450.t1.2	-2.66	0.011	Ubiquitin-conjugating enzyme E2 I (UBE2I, UBC9)
cre03.g167000.t1.2	-3.26	0.038	Ubiquitin-conjugating enzyme E2
cre07.g342506.t1.1	4.88	0.006	Ubiquitin-conjugating enzyme
g3389.t1	3.99	0.028	ubiquitin-like modifier-activating enzyme ATG7 (ATG7)

Supplementary Figure S5. The genes belonging to proteasome and autophagy according to the MapMan analysis of *Chlamydomonas reinhardtii* cells in response to 0.3 mM SNAP treatment for 1 h as compared to the control.

photosynthesis

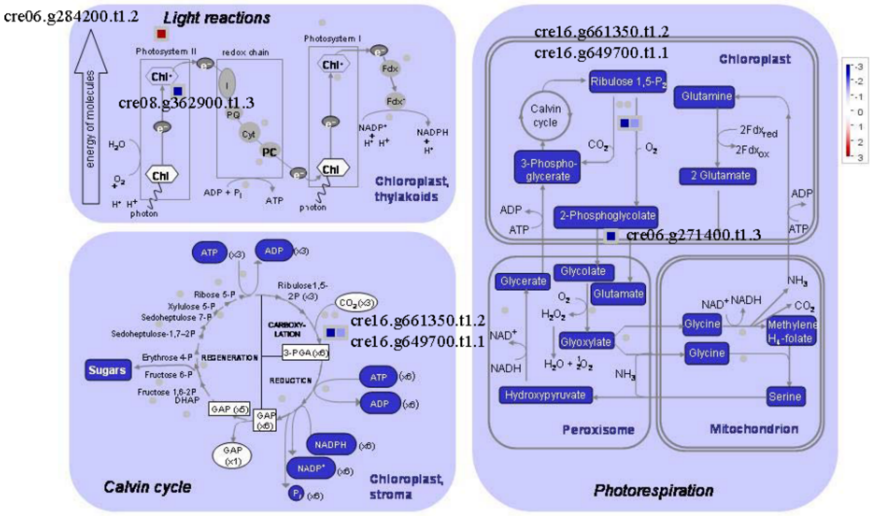

Gene	Fold Change (Log2)	P-value	Seq. description
cre06.g284200.t1.2	1.10	0.005	Chlorophyll *a/b* binding protein of LHCII
cre08.g362900.t1.3	-1.38	0.003	Lumenal PsbP-like protein
cre16.g661350.t1.2	-1.22	0.014	Rubisco large subunit N-methyltransferase
cre16.g649700.t1.1	-0.59	0.024	ribulose-bisphosphate carboxylase oxygenase large subunit n- chloroplastic-like
cre06.g271400.t1.3	-0.93	0.007	Phosphoglycolate phosphatase/4-nitrophenylphosphatase

Supplementary Figure S6. The genes belonging to photosynthesis (electron transport, Calvin cycle, and photorespiration) according to the MapMan analysis of *Chlamydomonas reinhardtii* cells in response to 0.3 mM SNAP treatment for 1 h as compared to the control.

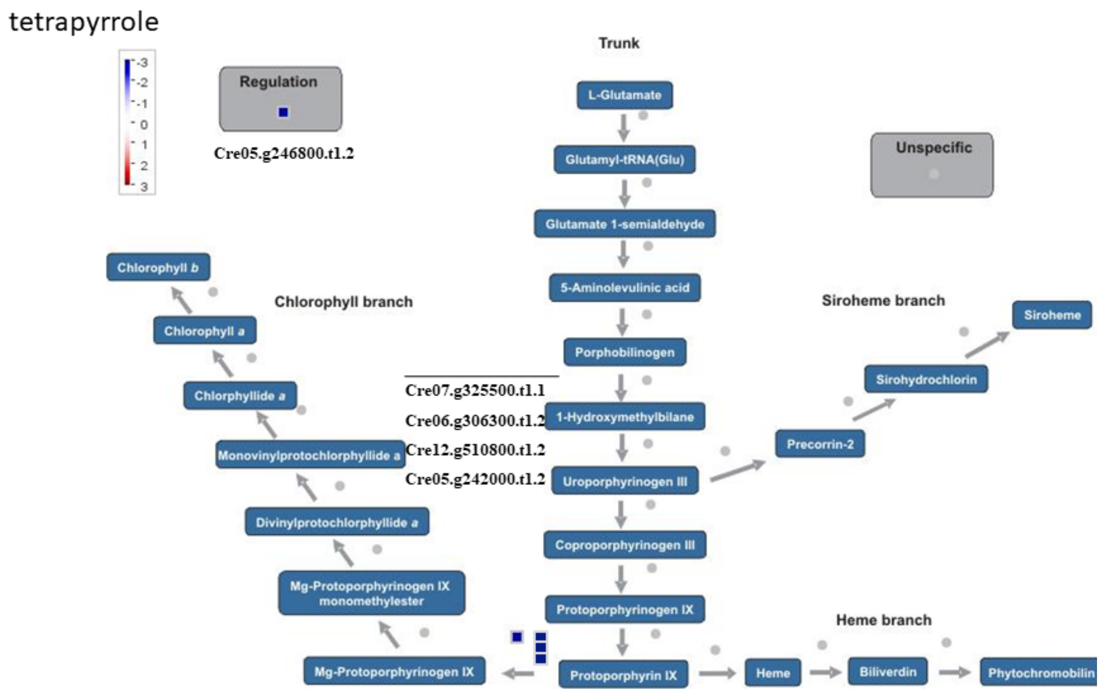

Gene	Fold Change (Log2)	P-value	Seq. description
Cre07.g325500.t1.1	-4.77	0.0128	Magnesium chelatase subunit H (CHLH)
Cre06.g306300.t1.2	-3.61	0.0261	Magnesium chelatase subunit I (CHLI1)
Cre12.g510800.t1.2	-5.61	0.0273	Magnesium chelatase subunit I (CHLI2)
Cre05.g242000.t1.2	-3.64	0.0191	Magnesium chelatase subunit D (CHLD)
Cre05.g246800.t.1.2	-7.38	0.0057	Tetrapyrrole-binding protein (GUN4)

Supplementary Figure S7. The genes belonging to tetrapyrrole pathway according to the MapMan analysis of *Chlamydomonas reinhardtii* cells in response to 0.3 mM SNAP treatment for 1 h as compared to the control.

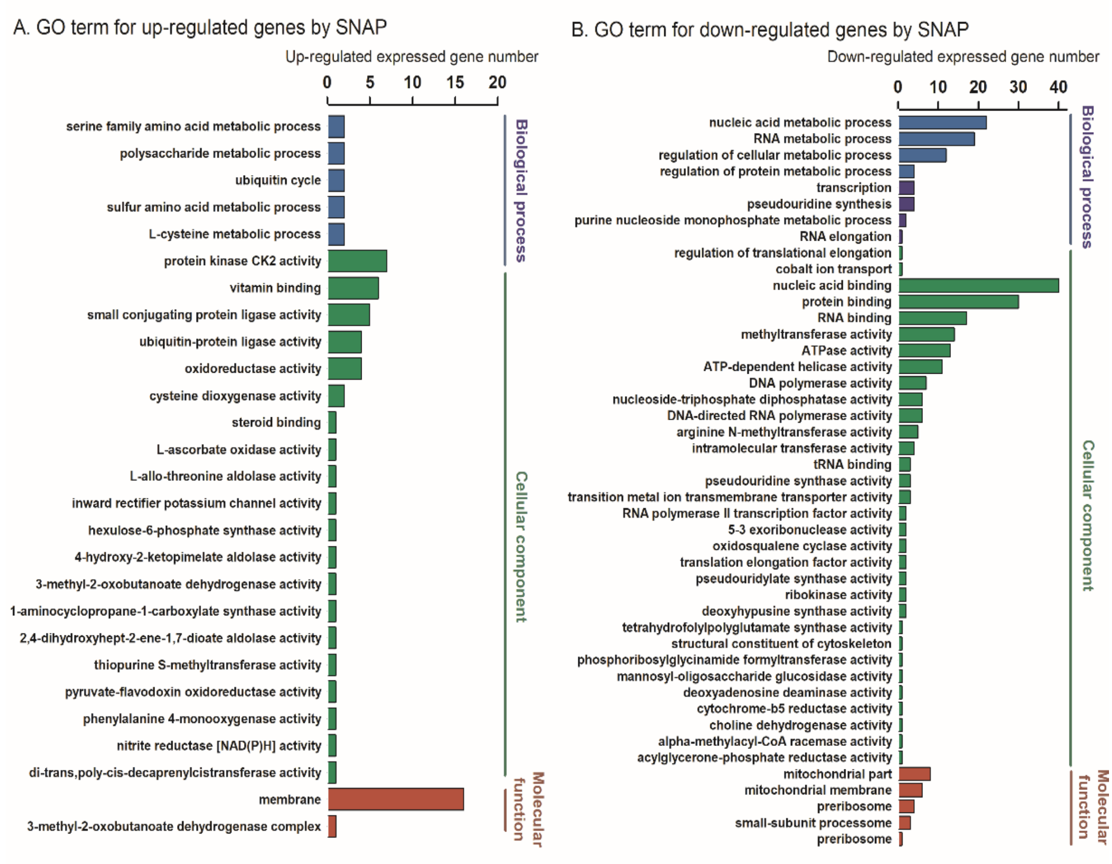

Supplementary Figure S7. GO terms of significantly upregulated (A) and downregulated (B) genes in *Chlamydomonas reinhardtii* cells in response to 0.3 mM SNAP treatment for 1 h as compared to the control.

Among the NO-upregulated genes, the GO terms for the 'Biological process' were L-cysteine metabolic process, sulfur amino acid metabolic process, serine family amino acid metabolic process, polysaccharide metabolic process, and ubiquitin cycle, while those for the 'Cellular component' were protein kinase CK2 activity, vitamin binding, small conjugating protein ligase activity, ubiquitin-protein ligase activity, oxidoreductase activity, cysteine dioxygenase activity, and so on (Supplementary Table S5A). The majority of the GO terms for the NO-upregulated genes in the 'Molecular function' was the membrane group. For the NO-downregulated genes, the majority of the GO terms for the NO-downregulated genes linked to transcriptional and translational regulation including nucleic acid, RNA, and protein metabolic process, pseudouridine synthesis, nucleic acid binding, protein binding, RNA binding, and mitochondrial part (Supplemental Table S5B).

Materials and Methods

cDNA Library Preparation, Illumina Sequencing, and Sequence Analysis

Total RNA was extracted using the TRIzol® Reagent (Invitrogen, Eugene, OR, USA) according to the manufacturer's protocol. To obtain complete gene expression information, RNA samples from algae cultured under different conditions were pooled for Illumina sequencing. Briefly, poly (A)+RNA was purified from 5 μg of pooled total RNA using oligo (dT) magnetic beads, sheared into short fragments, and primed for cDNA library synthesis using the TruSeq RNA sample preparation kit

according to the manufacturer's instructions (Illumina, San Diego, CA, USA). After quantitation using a TBS-380 minifluorometer (PicoGreen), the samples were clustered (TruSeq paired-end cluster kit v3-cBot-HS; Illumina, San Diego, CA, USA) and sequenced on the HiSeq2000 platform (100 bp, TruSeq SBS kit v3-HS 200 cycles; Illumina, San Diego, CA, USA) by 2 × 90 bp pair-end mode. The library construction and sequencing were performed by YOURGENE BIO-SCIENCE Company (New Taipei City, Taiwan).

mRNA molecules with poly-A tails were purified using poly-T oligo-attached magnetic beads from eukaryote (prokaryote can be treated with kit to remove rRNA before next step). The cDNA is synthesized by using random Hexamer priming. The second-strand is generated to create double-strand cDNA. The cDNA is purified by the use of Qiagen Purification Kit followed by performing end repair and A-tailing. After these procedures, the library could be sequenced using IlluminaHiSeq™. First step in the trim process is to convert the quality score (Q) to error probability. Next, for every base a new value is calculated: 0.05-Error probability. This value will be negative for low quality bases, where the error probability is high. For every base, we calculate the running sum of this value. If the sum drops below zero, it is set to zero. The part of the sequence to be retained is between the first positive value of the running sum and the highest value of the running sum. Everything before and after this region is trimmed off. In addition, if the read length is shorter than 35 bp, the read will be discarded. We then map trimmed reads of each sample to the reference by using gapped alignment and soft clip strategy to calculate gene expression. This method is essential for variant discovery because the sequence reads may contain insertion-deletion polymorphism (INDEL). Without this alignment approach, a read may still be mapped onto the correct position but with consecutive mismatches at INDEL locations. A read is mapped onto the reference if it has at least 80% similarity in matched region, and the matched region is at least 90% of the read length fraction. The original gene expression values often need to be transformed and/or normalized in order to ensure that samples are comparable and assumptions on the data for analysis are met (Allison et al., 2006). The expression level for each unigene was calculated and normalized using the RPKM (reads per kb per million reads; Mortazavi et al., 2008). Fragments per kilobase per million (FPKM), expected fragments per kilobase of transcript per million fragments sequenced, is defined according to Trapnell et al. (2010) as total fragments/mapped reads (millions)×exon length (KB). The total fragments are the number of paired-end reads that have been mapped to a region in which an exon is annotated for the gene or across the boundaries of two exons or an intron and an exon for an annotated transcript of the gene. The mapped reads (million) is the total number of reads that after mapping have been mapped to the region of the gene. The exon length (KB) is calculated as the sum of the lengths of all exons annotated for the gene, divided by 1,000. Each exon is included only once in this sum, even if it is present in more annotated transcripts for the gene. Besides, when counting the mapped reads to generate expression values, we need to decide how to handle paired reads. The standard behavior is this: if two reads map as a pair, the pair is counted as one. If the pair is broken, none of the reads are counted. The reasoning is that something is not right in this case, it could be that the transcripts are not represented correctly on the reference, or there are errors in the data. In general, more confidence is placed with an intact pair. An R/Bioconductor package with variance and mean linked by local regression and present an implementation, DESeq, to analyze the statistic test for differential expression by using the negative binomial

distribution to model the null distribution of the read count data (Anders and Huber, 2010), and only those genes with a FPKM value larger than one in any sample were retained for subsequent analyses. The negative binomial distribution is a generalization of the Poisson model that allows to model biological variance correctly. Differentially expressed genes (DEGs) were identified in control, SNAP, cPTIO, and 'SNAP+cPTIO' treatments according to the $|\log_2$ (fold-change)$| \geq 1.2$ with the false discover rate (FDR) *P*-value ≤ 0.05, and max FPKM ≥ 1). Whether SNAP effect was attributable to NO was examined by the treatment together with a NO scavenge, cPTIO. In addition, Gene Ontology (GO) Enrichment Analysis Software Toolkit were used to multi-GO enrichment analyses of DEGs (Zheng and Wang, 2008).

Gene annotations were retrieved from the Chlamydomonas genome browser in the U.S. Department of Energy (DOE) Joint Genome Institute (JGI) (http://genome.jgi-psf.org/Chlre3/Chlre3.home.html). For genes with no associated annotation, BLAST searches were performed to identify sequence similarities. Sequence analysis and alignment were performed using DNAstar software v.4.05 (Lasergene Navigator, Madison, WI, USA), Bioedit Sequence Alignment Editor version 5.0.9 (Department of Microbiology, North Carolina State University), and the National Center for Biotechnology Information BLAST server (http://www.ncbi.nlm.nih.gov/BLAST/).

Literature Cited

Allison, D. B., Cui, X., Page, G. P. and Sabripour, M. (2006). Microarray data analysis: From disarray to consolidation and consensus. *Nat. Rev. Genet.* 7, 55-65

Anders, S. and Huber, W. (2010). Differential expression analysis for sequence count data. *Genome Biol.* 11, R106

Mortazavi, A., Williams, B. A., McCue, K., Schaeffer, L. and Wold, B. (2008). Mapping and quantifying mammalian transcriptomes by RNA-Seq. *Nat. Methods* 5, 621-628 doi: 10.1038/nmeth.1226

Zheng, Q. and Wang, X. J. (2008). GOEAST: a web-based software toolkit for Gene Ontology enrichment analysis. *Nuc. Acids Re*s. 36, suppl W358-W363

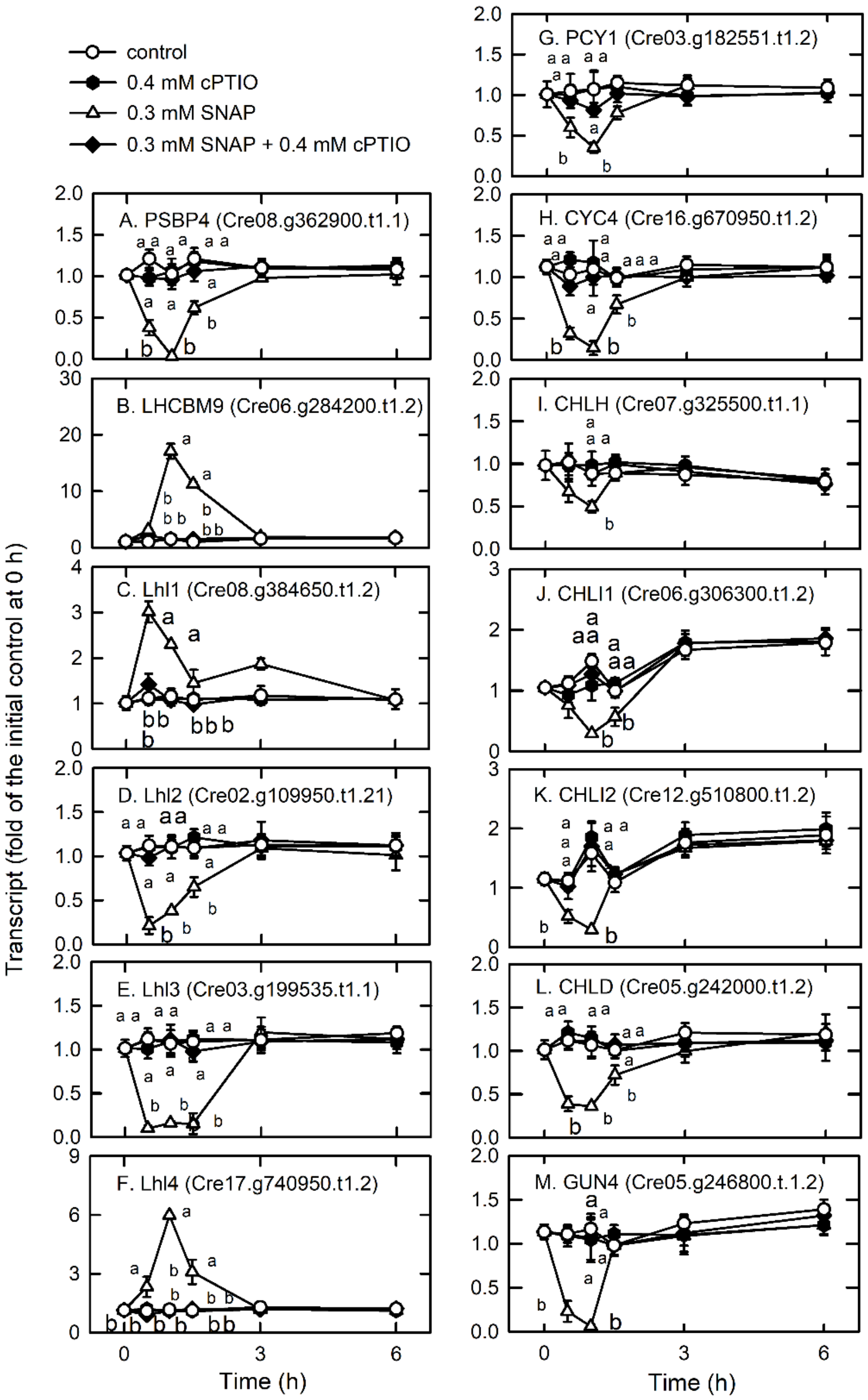

Supplementary Figure S9. **Effects of NO on the expression of genes encoding photosynthetic electron transport chain-related proteins.** Time-course changes in the transcript abundances of PSBP4 (A), LHCBM9 (B), Lhl1 (C), Lhl2 (D), Lhl3 (E), Lhl4 (F), PCY1 (G), CYC4 (H), CHLH (I), CHLI1 (J), CHLI2 (K), CHLD (L), and GUN4 (M) in C. reinhardtii upon exposure to 0.3 mM SNAP in the presence or absence of 0.4 mM cPTIO. Data are expressed as the mean ± SD (n = 3). Different symbols indicate significant differences between treatments (Scheffe’s test, P < 0.05).

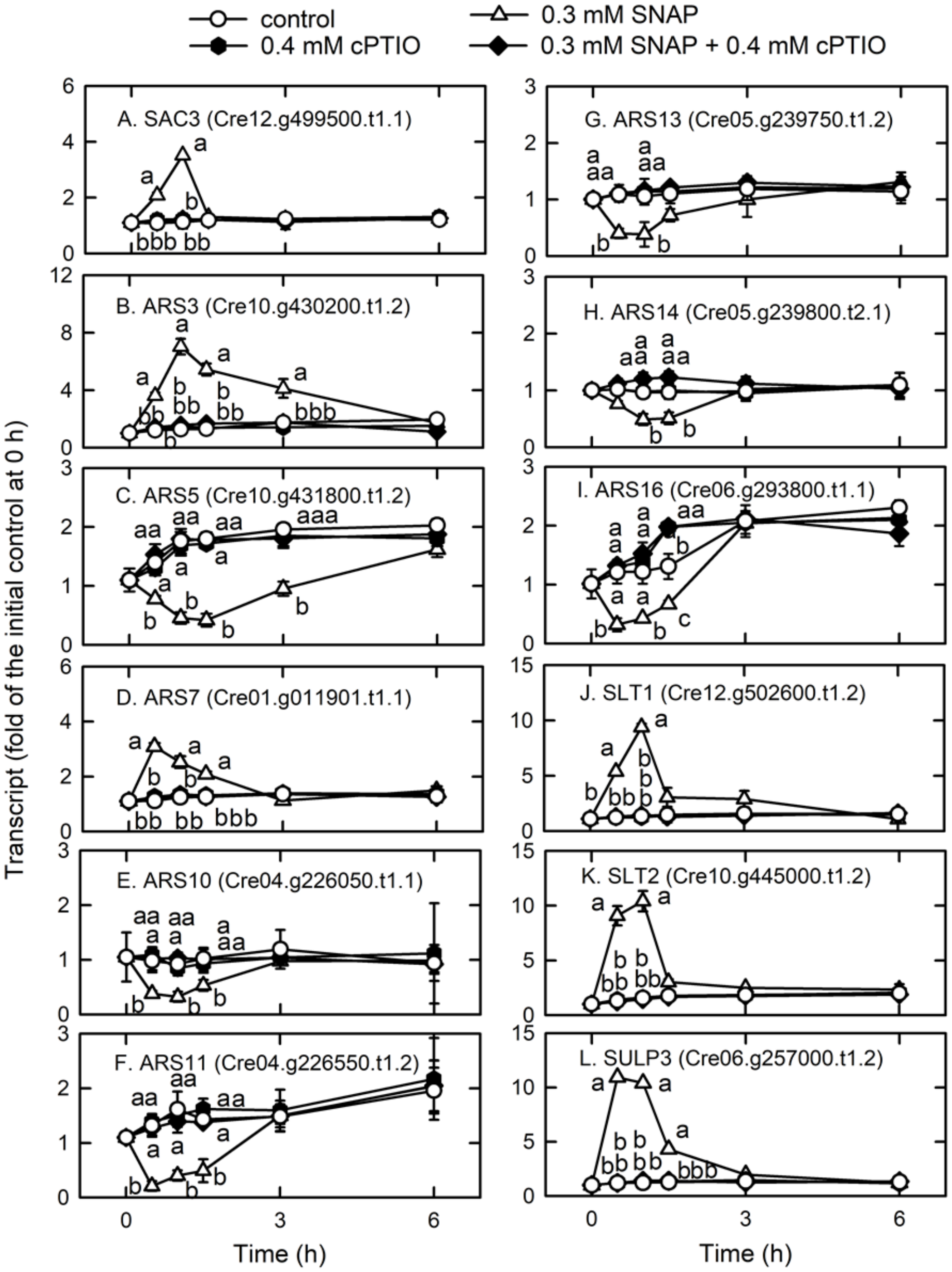

Supplementary Figure S10. Effects of NO on the expression of genes encoding proteins associated with sulfur availability. Time-course changes in the transcript abundances of SAC3 (A), SUOX (B), ARS3 (C), ARS7 (E), ARS5 (D), ARS11 (G), ARS13 (H), ARS14 (I), ARS16 (J), SLT1 (K), SLT2 (L), and SULP3 (M) in *C. reinhardtii* after exposure to 0.3 mM SNAP in the presence or absence of 0.4 mM cPTIO. Data are expressed as the mean ± SD (n = 3). Different symbols indicate significant differences between treatments (Scheffe's test, $P < 0.05$).

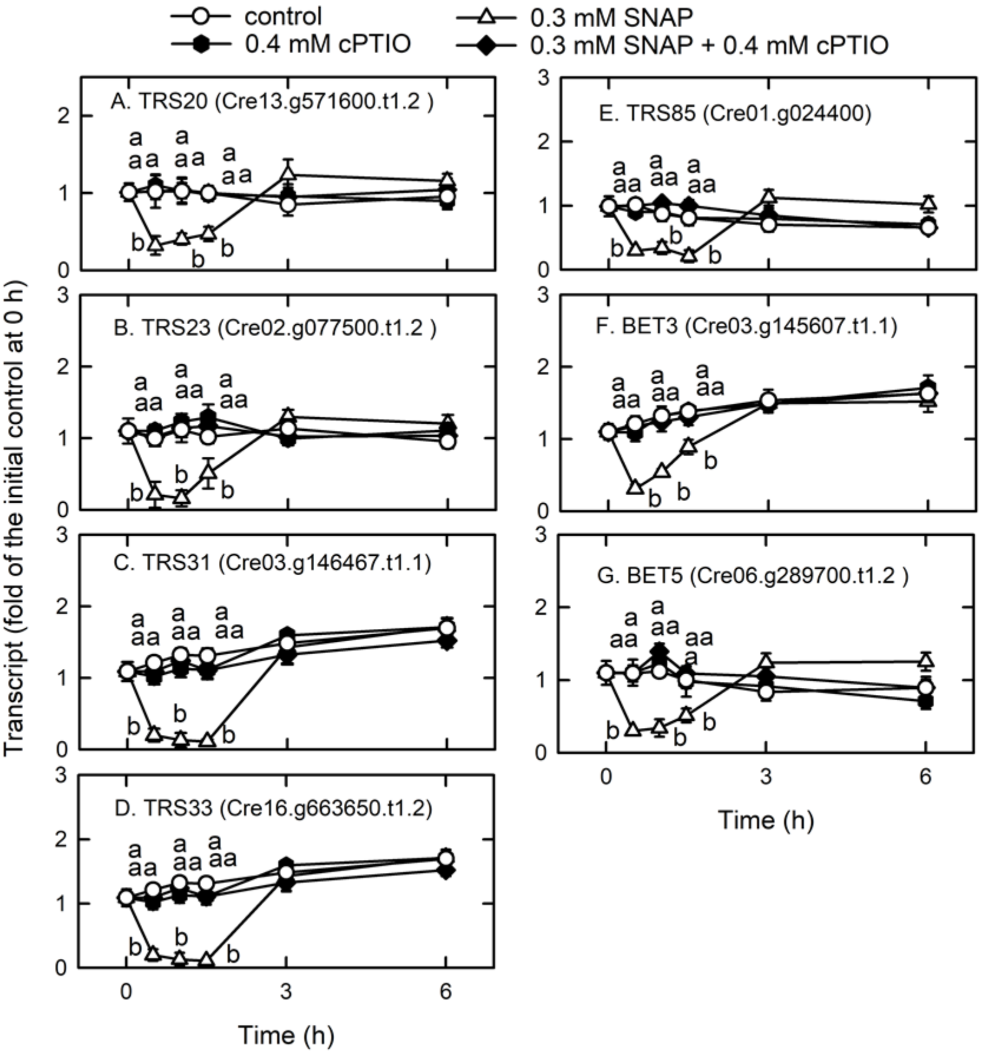

Supplementary Figure S11. Effects of NO on the expression of genes encoding proteins involving in protein glycosylation process. Time-course changes in the transcript abundances of TRS20 (A), TRS23 (B), TRS31 (C), TRS33 (D), TRS85 (E), BET3 (F), and BET5 (G) in *C. reinhardtii* after exposure to 0.3 mM SNAP in the presence or absence of 0.4 mM cPTIO. Data are expressed as the mean ± SD (n = 3). Different symbols indicate significant differences between treatments (Scheffe's test, $P < 0.05$).

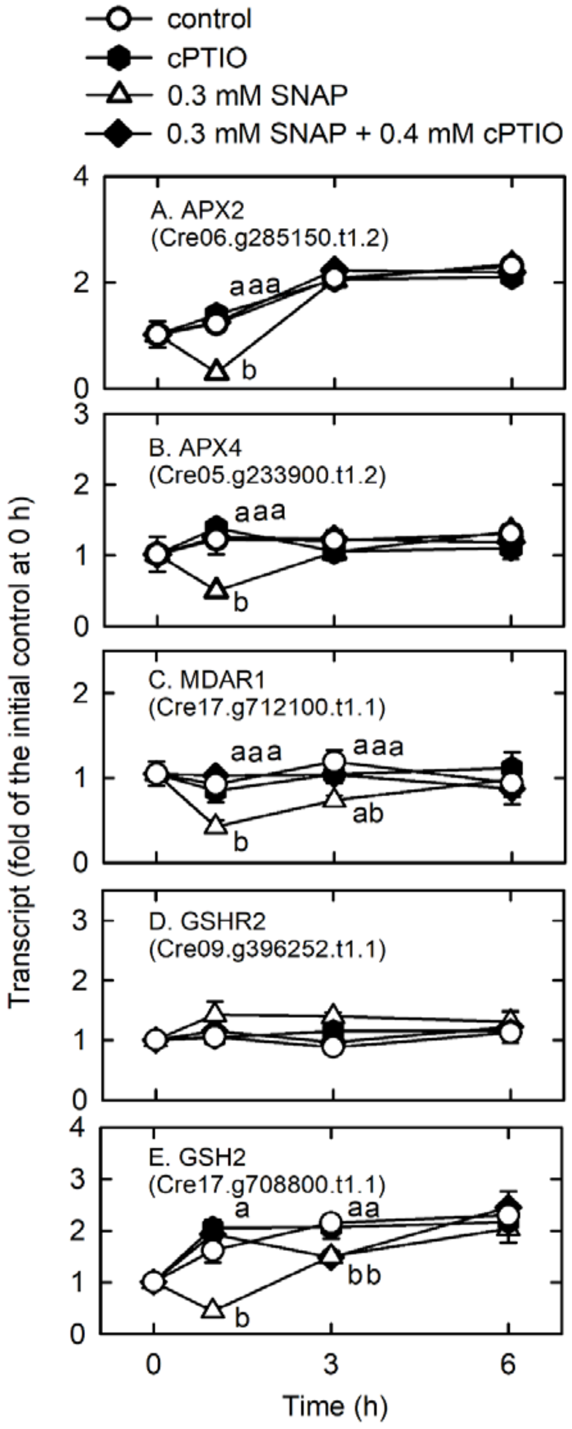

Supplementary Figure S12. Time-course changes in the transcript abundances of APX2 (Cre06.g285150.t1.2) (A), APX4 (Cre05.g233900.t1.2) (B), GSHR2 (Cre09.g396252.t1.1) (C), and GSH2 (Cre17.g708800.t1.1) (D) in *Chlamydomonas reinhardtii* upon exposure to 0.3 mM SNAP in the presence or absence of 0.4 mM cPTIO. Data are expressed as the mean ± SD (n = 3). Different symbols indicate significant differences between treatments at the same time (Scheffe test, $P < 0.05$).

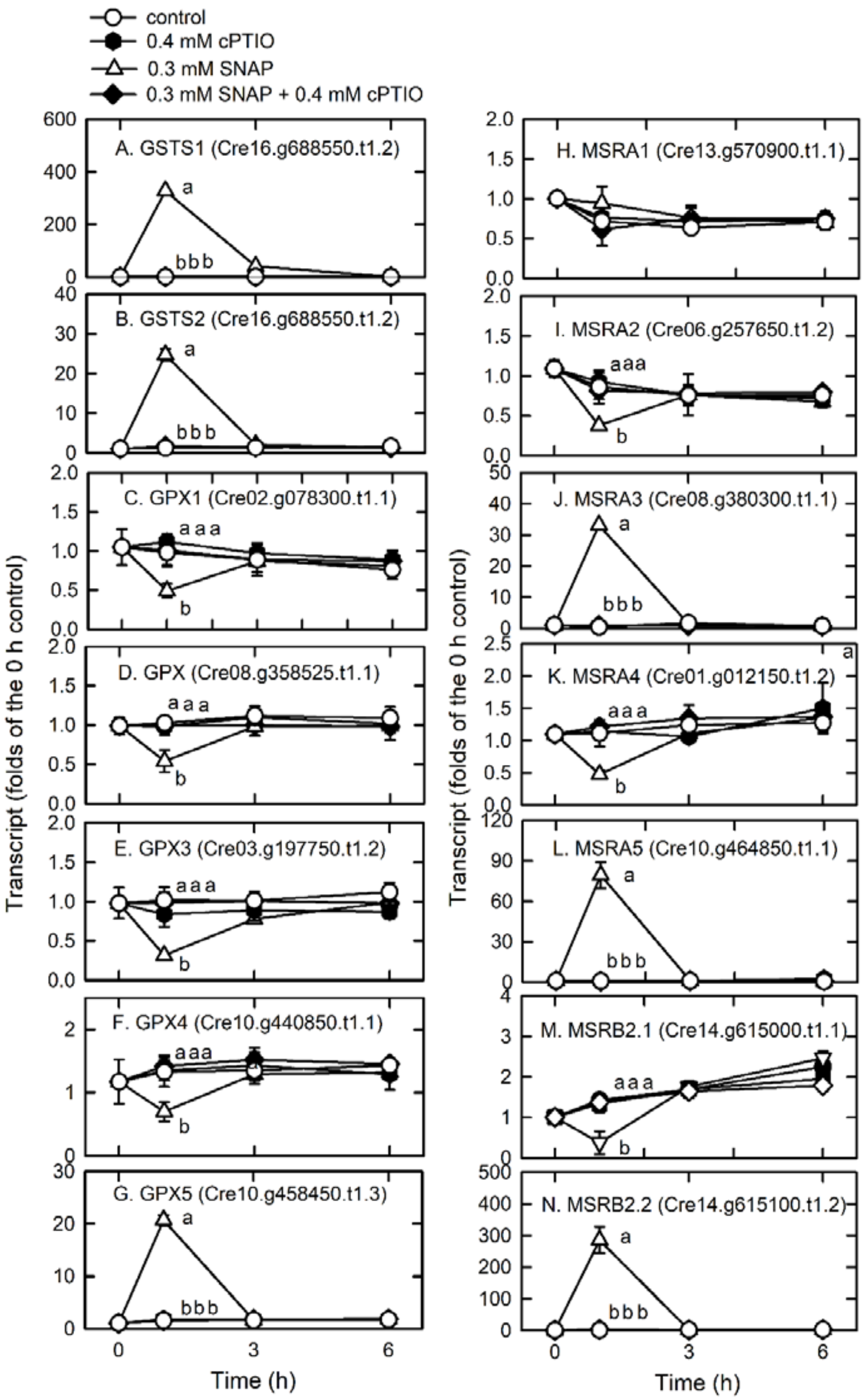

Supplementary Figure S13. Time-course changes in the transcript abundances of GPX1 (Cre02.g078300.t1.1) (A), GPX (Cre08.g358525.t1.1) (B), GPX3 (Cre03.g197750.t1.2) (C), GPX4 (Cre10.g440850.t1.1) (D), GPX5 (Cre10.g458450.t1.3) (E), MSRA1 (Cre13.g570900.t1.1) (F), MSRA2 (Cre06.g257650.t1.2) (G), MSRA3 (Cre08.g380300.t1.1) (H), MSRA4 (Cre01.g012150.t1.2) (I), MSRA5 (Cre10.g464850.t1.1) (J), MSRB2.1 (Cre14.g615000.t1.1) (K), and MSRB2.2 (Cre14.g615100.t1.2) (L) in *Chlamydomonas reinhardtii* upon exposure to 0.3 mM SNAP in the presence or absence of 0.4 mM cPTIO. Data are expressed as the mean ± SD (n = 3). Different symbols indicate significant differences between treatments at the same time (Scheffe test, $P < 0.05$).

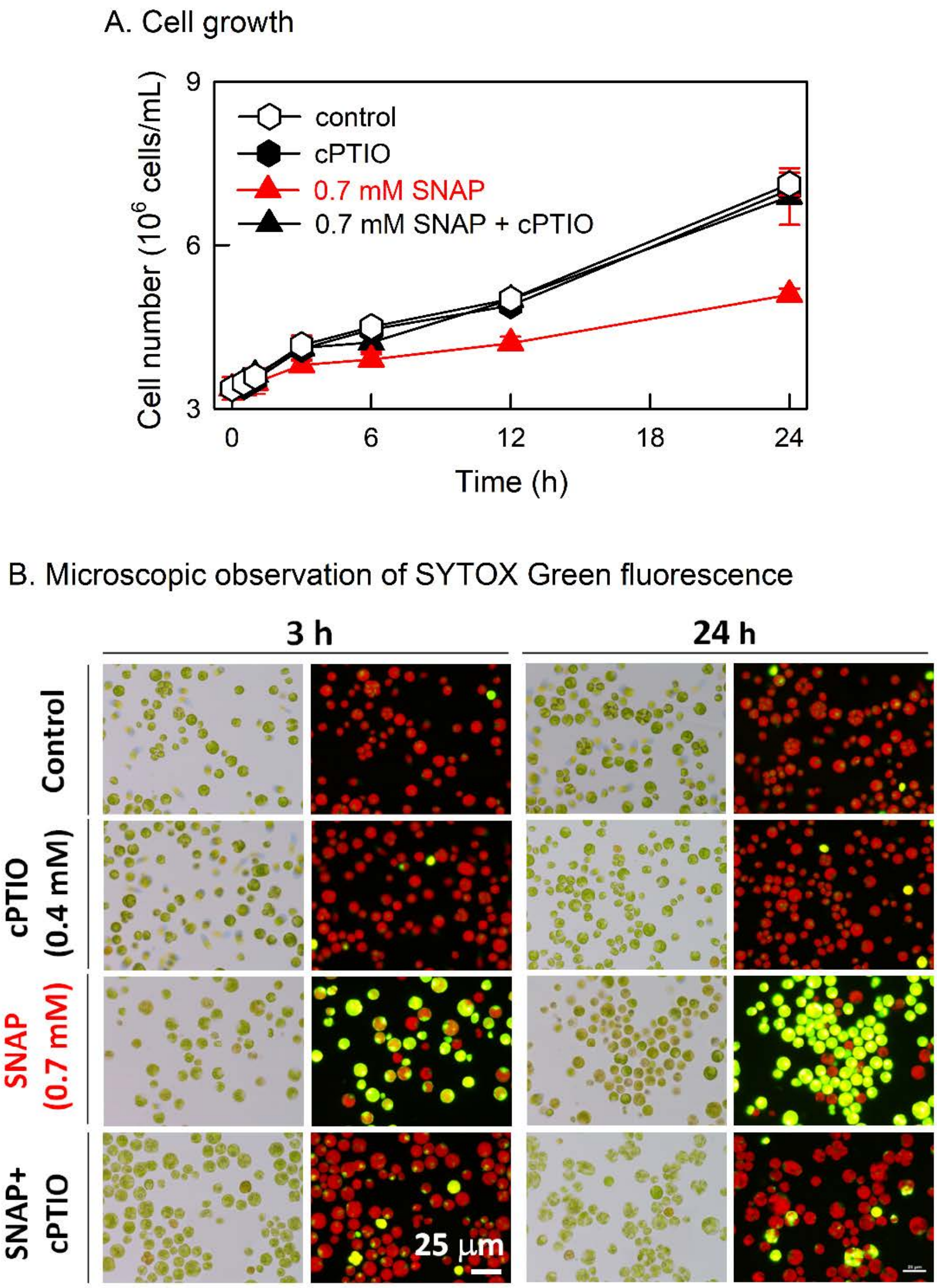

Supplementary Figure S14. Physiological response to 0.7 mM SNAP treatment (sub-lethal NO stress) in *Chlamydomonas reinhardtii* in the presence or absence of 0.4 mM cPTIO. A, Cell growth; B, Cell death assessed by SYTOX green fluorescence. Data are expressed as the mean ± SD (n = 3). Different symbols indicate significant differences between treatments (Scheffe's test, $P < 0.05$).

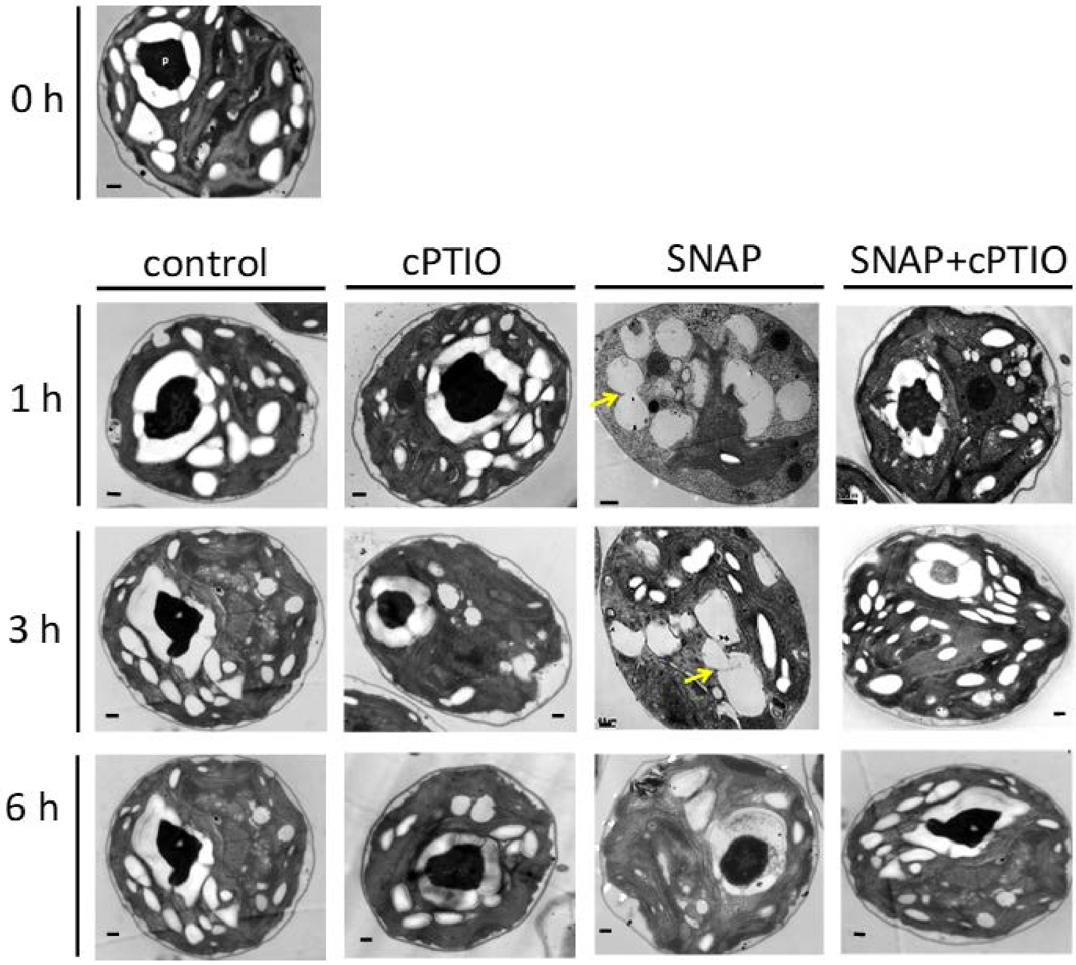

Supplementary Figure S15. Transmission electron microscopic observation of *Chlamydomonas reinhardtii* upon exposure to 0.3 mM SNAP in the presence or absence of 0.4 mM cPTIO. The yellow arrow indicates the fusion of two vesicles. Black bar indicates 0.5 μm.

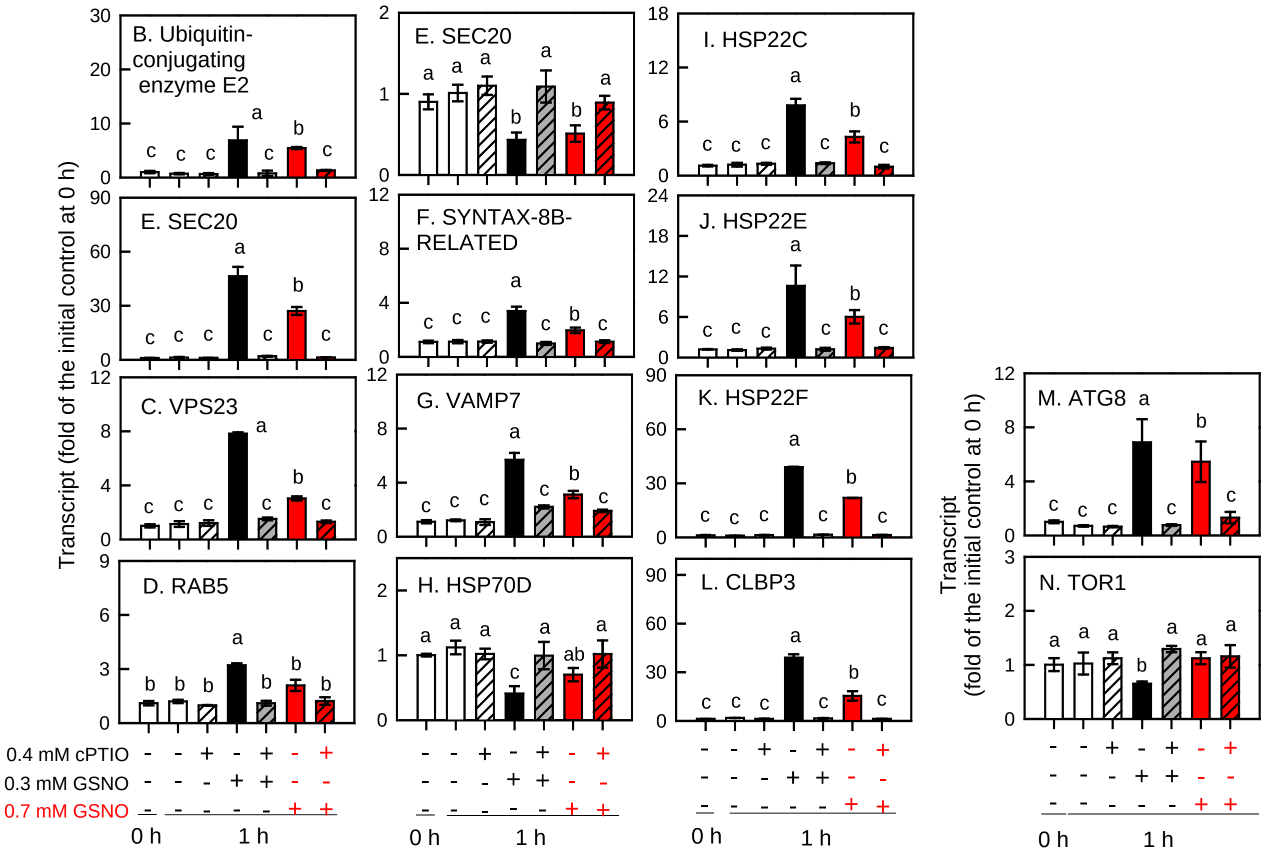

Supplementary Figure S16. Transcript abundances of ubiquitin-conjugating enzyme E2 (A), UBE3A (B), VPS23 (C), RAB5 (D), SEC20 (E), SYNTAX-8B-RELATED (F), VAMP7 G), HSP70D (H), HSP22C (I), HSP22E (J), HSP22F (K), CLBP3 (L), ATG8 (M), and TOR1 (N), in *Chlamydomonas reinhardtii* upon exposure to 0.3 or 0.7 mM GSNO in the presence or absence of 0.4 mM cPTIO. Data are expressed as the mean ± SD (n = 3). Different symbols indicate significant differences between treatments at the same time (Scheffe test, $P < 0.05$).

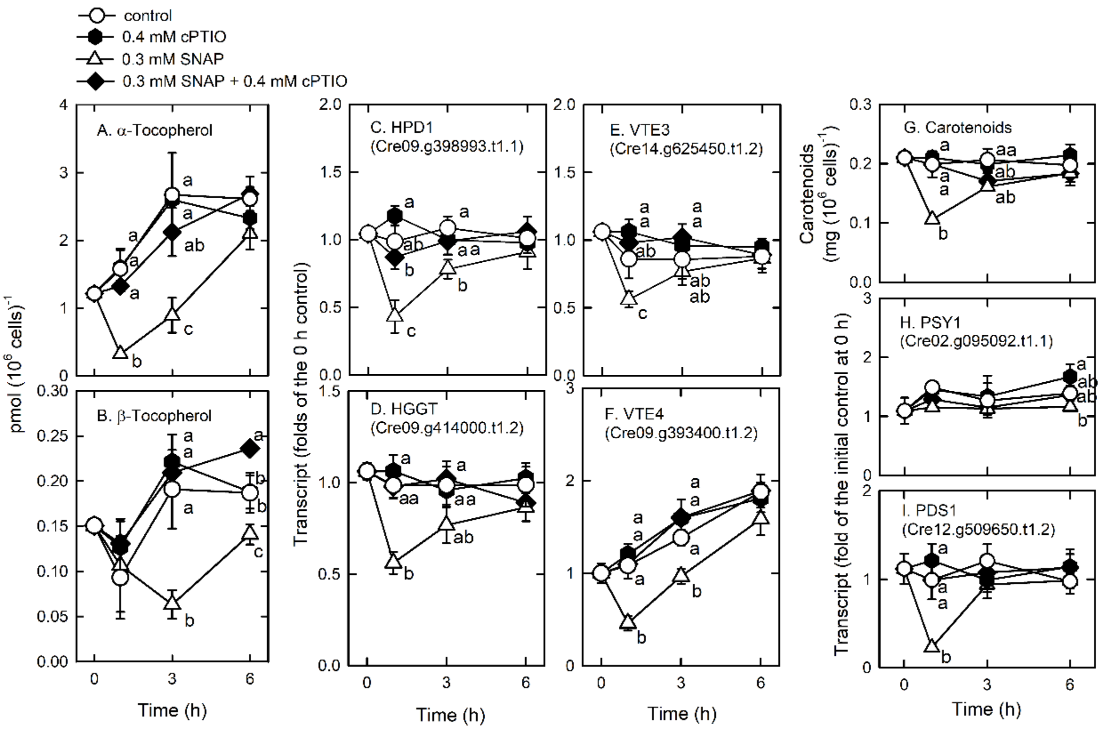

Supplementary Figure S17. Time-course changes in the concentrations of α-tocopherol (A) and β-tocopherol (B) and the transcript abundances of HPD1 (Cre09.g398993.t1.1) (C), HGGT (Cre09.g414000.t1.2) (D), VTE3 (Cre14.g625450.t1.2) (E), and VTE4 (Cre09.g393400.t1.2) (F), the concentrations of carotenoids (G) and the transcript abundances of PSY1 (Cre02.g095092.t1.1) (H) and PDS1 (Cre12.g509650.t1.2) (I) in *Chlamydomonas reinhardtii* upon exposure to 0.3 mM SNAP in the presence or absence of 0.4 mM cPTIO. Data are expressed as the mean ± SD (n = 3). Different symbols indicate significant differences between treatments at the same time (Scheffe test, $P < 0.05$).

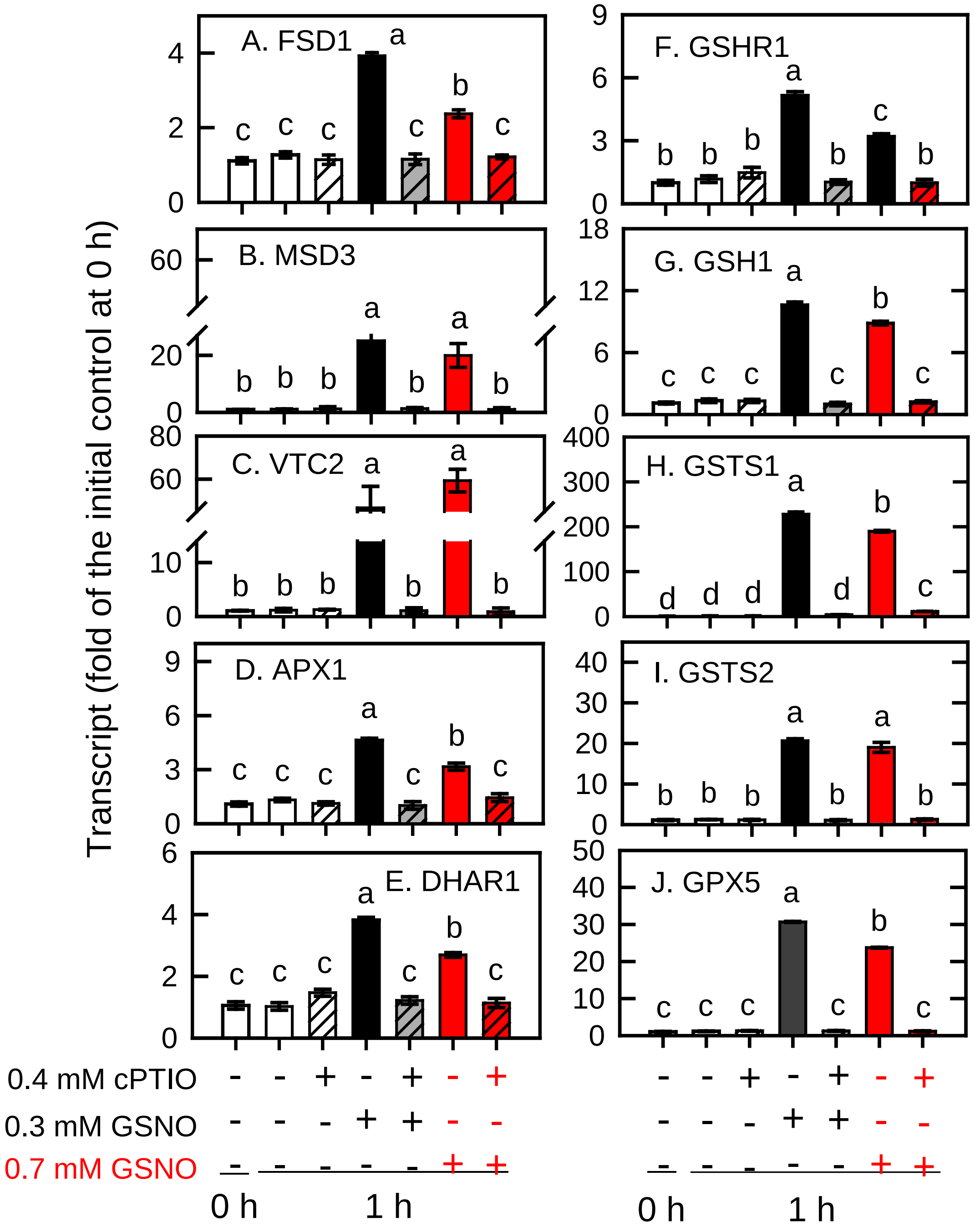

Supplementary Figure S18. Transcript abundances of FSD1 (A), MSD3 (B), VTC2 (C), APX1 (D), DHAR1 (E), GSHR1 (F), GSH1 G), GSTS1 (H), GSTS2 (I), and GPX5 in *Chlamydomonas reinhardtii* upon exposure to 0.3 or 0.7 mM GSNO in the presence or absence of 0.4 mM cPTIO. Data are expressed as the mean ± SD (n = 3). Different symbols indicate significant differences between treatments at the same time (Scheffe test, $P < 0.05$).
